# Pullulan nanoparticles inhibit the pathogenicity of *Candida albicans* by regulating hypha-related gene expression

**DOI:** 10.1128/spectrum.01048-24

**Published:** 2024-11-14

**Authors:** Sujin Hong, Seo-Kyung Kim, Christine H. Chung, Cheol-Heui Yun, Junho Lee, Chong-Su Cho, Won-Ki Huh

**Affiliations:** 1 School of Biological Sciences, Seoul National University, Seoul, Republic of Korea; 2 Institute of Microbiology, Seoul National University, Seoul, Republic of Korea; 3 Department of Agricultural Biotechnology, Seoul National University, Seoul, Republic of Korea; 4 Institute of Molecular Biology and Genetics, Seoul National University, Seoul, Republic of Korea; 5 Interdisciplinary Graduate Program in Genetic Engineering, Seoul National University, Seoul, Republic of Korea; 6 Research Institute of Agriculture and Life Sciences, Seoul National University, Seoul, Republic of Korea; Universidade de Brasilia, Brasilia, Brazil

**Keywords:** *Candida albicans*, pullulan nanoparticles, biofilm formation, pathogenicity, hypha-related gene

## Abstract

*Candida albicans* is a prevalent opportunistic pathogenic fungus that resides in the skin and gastrointestinal (GI) tract of humans. Under specific conditions, *C. albicans* cells transition from a commensal to a pathogenic state, leading to both superficial and invasive infections. Although systemic candidiasis poses a life-threatening risk, a limited number of antifungal drugs are employed for its treatment. Moreover, the emergence of resistant strains to antifungal agents underscores the pressing need for new treatment options. In this study, we propose the use of polysaccharide nanoparticles as a strategy for treating candidiasis. We synthesized phthalic pullulan nanoparticles (PPNPs) and examined their ability to inhibit the pathogenicity of *C. albicans*. We observed that PPNPs inhibit hyphal growth, adhesion to abiotic surfaces, and biofilm formation of *C. albicans* in a dose-dependent manner. This inhibitory effect is mediated by transcriptional modulation, particularly the downregulation of hypha-related genes and the upregulation of stress-responsive genes, involving the Ras/cAMP/PKA signaling pathway. Furthermore, we observed that PPNPs inhibit the adhesion of *C. albicans* to human epithelial cells without inducing toxicity in human cells. In addition, PPNPs inhibited the *in vivo* pathogenicity of *C. albicans* in *Caenorhabditis elegans*, suggesting an antagonistic effect on candidiasis. Our findings suggest that PPNPs exhibit inhibitory effects on *C. albicans* biofilm formation and *in vivo* pathogenicity, indicating their potential as a novel therapeutic agent for candidiasis.

**IMPORTANCE:**

The pathogenic process of *Candida albicans*, the primary causative species of candidiasis, involves hyphal growth, biofilm formation, and secretion of virulence factors. Of these factors, the biofilm, created by the secretion of extracellular matrix from adherent cells, shields cells from external threats, enabling them to withstand high concentrations of antifungal agents. Therefore, suppressing biofilm formation is a crucial aspect of combating candidiasis. This study developed phthalic pullulan nanoparticles (PPNPs) as a novel material for inhibiting *C. albicans*’ pathogenicity. PPNPs were internalized within *Candida* cells and reduced pathogenicity at the gene expression level, resulting in reduced *in vitro* biofilm formation, adhesion to human cells, and mortality of infected *Caenorhabditis elegans*. Moreover, PPNPs exhibited these effects without toxicity to human cells and host animals. These findings not only indicate that PPNPs can be employed to hinder *in vitro* biofilm formation but also suggest their potential as a novel treatment for candidiasis.

## INTRODUCTION

*Candida albicans* is the most common opportunistic fungus in the human body. Although it is normally commensal in the gastrointestinal (GI) tract and skin, it can cause disease in immunocompromised or medically compromised patients receiving antibiotic treatment (1). The diseases range from superficial candidiasis occurring in the vagina, mouth, or skin to systemic candidiasis that disseminates into various organs (2). Among them, systemic candidiasis is a life-threatening disease, with a mortality rate of 40% to 60% (2, 3). However, only a few antifungal drugs are mainly used for treatment (4). Nonetheless, treatment using antifungal agents has limitations due to their side effects, potential toxicity to humans, and the occurrence of resistant fungal strains (5–9). Therefore, the need for a new approach to treating candidiasis is emerging.

The primary species causing candidiasis is *C. albicans*, a dimorphic fungus with a rounded yeast form and filamentous hyphal and pseudo-hyphal forms (10, 11). The morphological transition, known as the “yeast-to-hypha transition,” is crucial for the pathogenicity of *C. albicans*. This transition is accompanied by filamentation, the expression of adhesive cell wall proteins, and the secretion of proteases, toxins, and extracellular matrix (12–14). The initial step in the pathogenic mechanism is attachment. *C. albicans*, when attached to biotic or abiotic surfaces, proliferates to form microcolonies that subsequently develop into mature biofilms through filamentous growth and extracellular matrix secretion. Particularly, this extracellular matrix serves as a barrier, protecting *Candida* cells from antifungal drugs, toxins, and phagocytic cells (15–18). In addition, mature biofilms can disperse, enabling *C. albicans* to colonize new locations (19, 20). As a result, candidiasis caused by *C. albicans* biofilm in medical devices such as intravascular catheters, dental implants, and artificial joints is recognized as a significant cause of hospital-acquired infections and exhibits severe antifungal resistance (21–23). Because all these processes can be regulated through the yeast-to-hypha transition, it is widely accepted that this transition in *C. albicans* is pivotal for the regulation of commensalism and pathogenicity. Despite not being extensively studied yet, inhibiting this transition has emerged as an alternative treatment for candidiasis (24–26).

Various environmental signals, such as temperature (27), nutrients (28), serum (29), pH (30), CO_2_ (31), and small molecules of bacterial origin (32), are known to induce the yeast-to-hypha transition. This transition occurs through the activation of hypha-specific gene expression via signal transduction pathways, such as the Ras/cAMP/PKA signaling pathway. Representative hypha-related genes include cell wall adhesin genes like *HWP1, ALS* adhesins*,* and the secreted protease genes *SAPs* (33–37). Previous studies have reported that inhibiting the yeast-to-hypha transition by suppressing the expression of hypha-specific genes can efficiently inhibit the pathogenicity of *C. albicans* (13, 14).

Polymeric nanoparticles (NPs), colloid solids with a size ranging from 10 to 1,000 nm, have been widely utilized in biomedical applications, such as drug delivery (38), gene delivery (39), vaccine delivery (40), and tissue engineering (41). They enable the transportation of bioactive agents into mammalian cells through the endocytosis mechanism, enhance the solubility of the hydrophobic drugs, prolong the circulation period of administered drugs in the blood, and can actively or passively target drugs to specific cells (42). In our previous studies (43–45), we observed that polysaccharide-based NPs are internalized by several lactic acid bacteria in the GI tract, enhancing the production of antibacterial polypeptides. Additionally, treatment with lactic acid bacteria and polysaccharide-based NPs altered the population of gut microbiome of mice *in vivo*, specifically increasing species diversity and the number of beneficial bacteria while decreasing the number of harmful bacteria (46, 47). These findings suggest that polysaccharide-based NPs can function as nano-prebiotics, promoting the growth and activity of probiotics. These results also prompt questions about the physiological impact of polysaccharide NPs on other organisms.

In this study, we synthesized phthalic pullulan (PP)-based NPs, referred to as PPNPs, and found that they inhibit the hyphal growth of *C. albicans* cells. PPNPs also inhibited the adhesion and biofilm formation of *C. albicans* on plastic surfaces. Additionally, we confirmed that PPNPs suppressed the transcription of hypha-related genes. Moreover, PPNPs exhibited an inhibitory effect on the adhesion of *C. albicans* to various human epithelial cells. The antagonistic effect of PPNPs on the *in vivo* pathogenicity of *C. albicans* was verified using the *Caenorhabditis elegans* infection model. Taken together, our findings suggest that PPNPs can provide a novel approach to treating candidiasis.

## RESULTS

### Characterization of PPNPs

**Fig 1:**
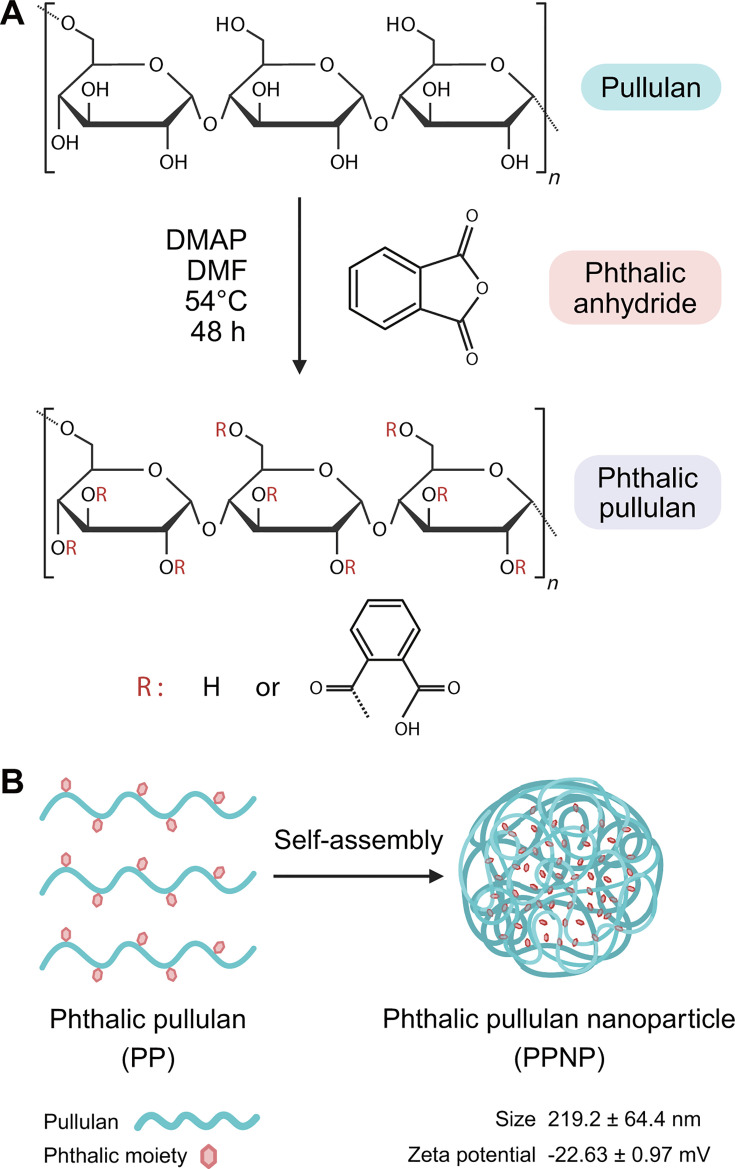
Synthesis of PPNPs. (**A**) Chemical reaction scheme for the synthesis of PP. (**B**) Formation of PPNPs through self-assembly of PP in aqueous solution. Size and zeta potential values represent the mean ± standard deviation. DMAP, dimethylaminopyridine; DMF, dimethylformamide.

The reaction scheme depicting the synthesis of PP is illustrated in Fig. 1A. Hydroxyl groups in the pullulan and carboxylic acid groups in the phthalic anhydride reacted in an organic solvent to form phthalic pullulan through an esterification reaction. To determine the proportion of phthalic groups incorporated into the phthalic pullulan, the degree of substitution of phthalic moieties within the pullulan was assessed via ^1^H-NMR spectroscopy (Fig. S1A). The peak assigned to the aromatic protons of phthalic acid appeared at 7.4–7.7 ppm, and the peak assigned to the anomeric protons of pullulan appeared at 4.68 and 5.00 ppm. Subsequently, the degree of substitution was calculated by evaluating the ratio of phthalate protons to pullulan protons, as previously described (48). As illustrated in Figure S1A, the content of phthalic groups per 100 glucose units of pullulan equaled 39%.

Upon dispersion of PP in an aqueous solution, it undergoes self-assembly through the hydrophobic interactions of the phthalic moieties (Fig. 1B). To investigate the characteristics of the self-assembled PPNPs dispersed in water, we analyzed the surface morphology of PPNPs using field-emission scanning electron microscope (FE-SEM). PPNPs were observed to take the form of nanometer-sized spheres (Fig. S1B). The size and zeta potentials of PPNPs in water were subsequently determined via dynamic light scattering (DLS) and electrophoretic light scattering (ELS), revealing a size of 219.2 ± 64.4 nm and a zeta potential of −22.63 ± 0.97 mV (Fig. S1C and D).

### PPNPs inhibit the hyphal growth of *C. albicans* cells

**Fig 2:**
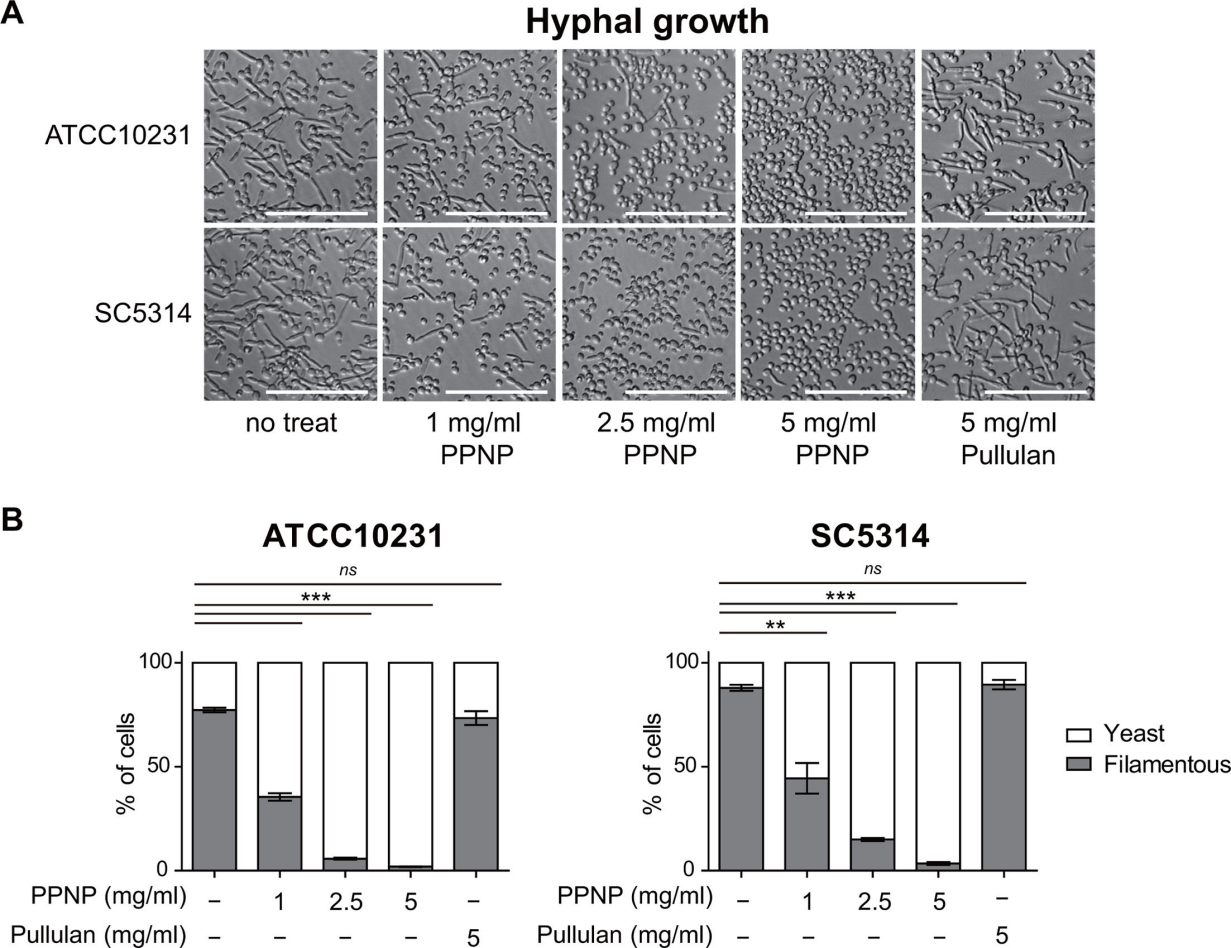
PPNPs inhibit the hyphal growth of *C. albicans*. (**A**) Morphology of *C. albicans* ATCC10231 cells and SC5314 cells observed by DIC microscopy. (**B**) Calculated proportion of yeast cells and filamentous (hypha and pseudohypha) cells. Cells were incubated in SC media containing 10% FBS with the indicated concentrations of PPNPs or pullulan at 37°C for 2 h. Asterisks indicate significant differences (paired two-tailed Student’s *t*-test): **P < 0.01; ***P < 0.001; *ns*, not significant. Scale bars, 50 µm.

To investigate the physiological effects of PPNPs on *C. albicans* cells, we first examined their impact on the growth of *C. albicans* cells. Our observation revealed that treatment with PPNPs did not affect the growth of *C. albicans* ATCC10231 cells at 37°C (Fig. S2). Next, we investigated the effect of PPNPs on the pathogenicity of *C. albicans*, focusing on the yeast-to-hypha transition, a key factor contributing to *C. albicans* pathogenicity (12, 49). We tested whether PPNPs affect the hyphal growth of *C. albicans* cells. As shown in Fig. 2A, when *C. albicans* ATCC10231 cells were cultured for 1 h at 37°C in synthetic complete (SC) medium supplemented with 10% fetal bovine serum (FBS), the majority of cells (approximately 77%) exhibited filamentous morphology, indicative of hyphal growth. Notably, treatment with 1 mg/mL of PPNPs reduced the proportion of filamentous cells to approximately 35% (Fig. 2B). Increasing the PPNP concentration to 2.5 and 5 mg/mL further decreased the proportion of filamentous cells to 6% and 2%, respectively. Pullulan, composed of maltotriose as a repeating unit, is produced by the fungus *Aureobasidium pullulans* (50). Because pullulan itself is a fungal metabolite, it is possible that the decrease in hyphal growth of *C. albicans* cells upon treatment with PPNPs is due to the presence of pullulan. To investigate this, we examined whether pullulan has an inhibitory effect on the hyphal growth of *C. albicans* cells. Unlike PPNPs, 5 mg/mL of pullulan did not inhibit the hyphal growth of *C. albicans* ATCC10231 cells.

To determine whether the inhibitory effect of PPNPs on the hyphal growth of *C. albicans* cells is specific to certain strains, we also monitored the hyphal growth of the SC5314 strain, a representative clinical isolate of *C. albicans*. Similar to ATCC10231 cells, PPNPs exhibited a dose-dependent inhibitory effect on the hyphal growth of SC5314 cells, while pullulan showed no inhibitory effect (proportion of filamentous cells: no treatment, 88%; 1 mg/mL PPNPs, 44%; 2.5 mg/mL PPNPs, 15%; 5 mg/mL PPNPs, 4%; 5 mg/mL pullulan, 90%) (Fig. 2A and B). These results suggest that PPNPs inhibit hyphal growth in *C. albicans* without affecting vegetative growth. This inhibitory effect is unique to PPNPs, not pullulan, and is consistent across different *C. albicans* strains.

### PPNPs inhibit the adhesion of *C. albicans* cells to abiotic surfaces

**Fig 3:**
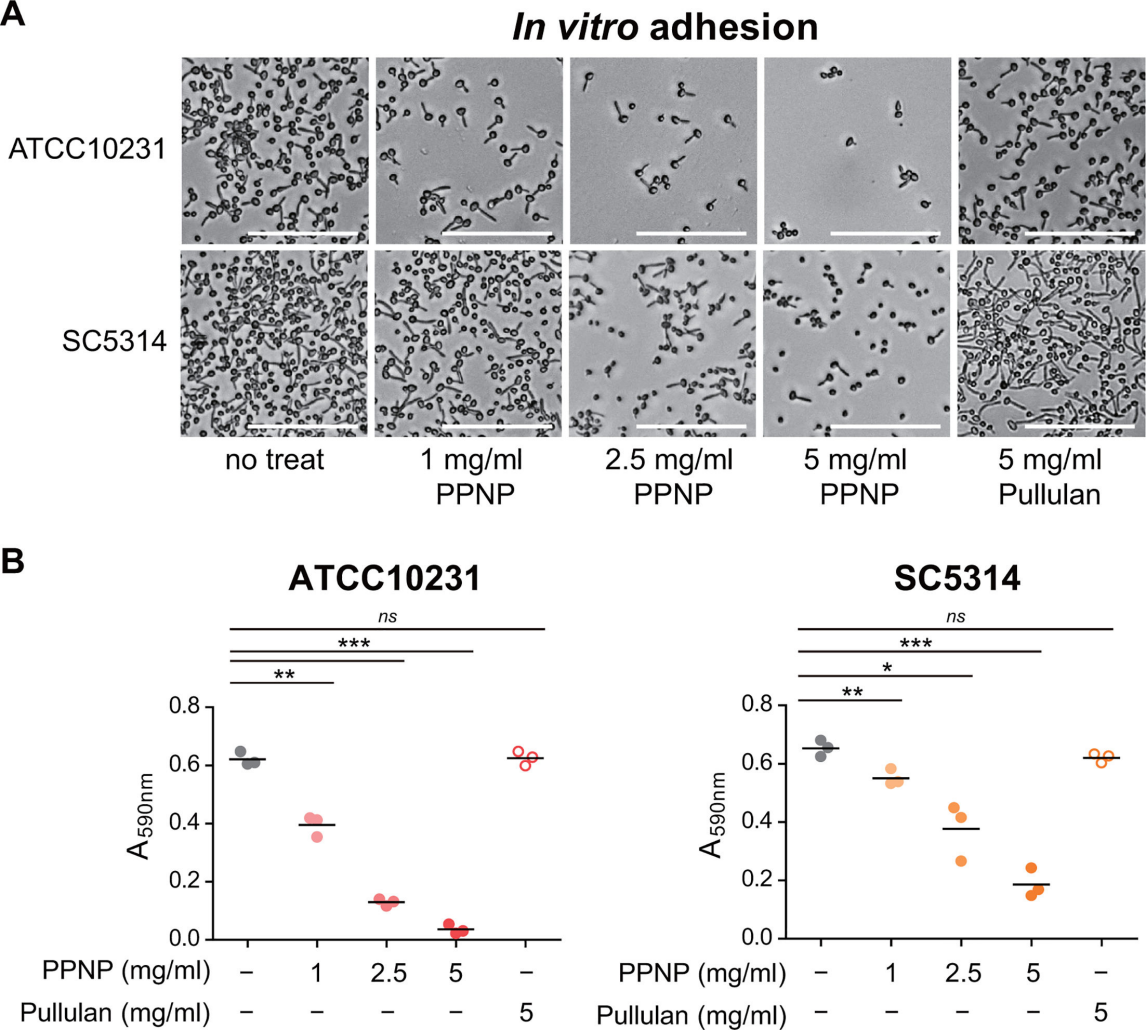
PPNPs inhibit *C. albicans* adhesion to abiotic surfaces. (**A**) Adherent cells observed by DIC microscopy. (**B**) Quantification of adherent cells by crystal violet staining. *C. albicans* ATCC10231 cells and SC5314 cells were incubated in SC media containing 10% FBS with the indicated concentrations of PPNPs or pullulan at 37°C for 1 h. Asterisks indicate significant differences (paired two-tailed Student’s *t*-test): *P < 0.05; **P < 0.01; ***P < 0.001; *ns*, not significant. Scale bars, 50 µm.

Considering that PPNPs inhibited the hyphal growth of *C. albicans* at concentrations above 1 mg/mL, they may also impede adhesion to abiotic surfaces, another key trait required for *C. albicans* pathogenicity. To test this hypothesis, we conducted an *in vitro* adhesion assay using the two *C. albicans* strains mentioned above. As shown in Fig. 3A, *C*. *albicans* cells efficiently adhered to the surfaces of cell culture plates after 1 h of incubation at 37°C in SC medium supplemented with 10% FBS. Consistent with the hyphal growth results, PPNPs inhibited the adhesion of *C. albicans* to abiotic surfaces in a dose-dependent manner (Fig. 3B). Additionally, 5 mg/mL of pullulan did not exhibit any inhibitory effect on adhesion to abiotic surfaces. These results suggest that PPNPs, not pullulan, inhibit cell adhesion to abiotic surfaces, regardless of the *C. albicans* strain.

### PPNPs inhibit the biofilm formation of *C. albicans* cells

**Fig 4:**
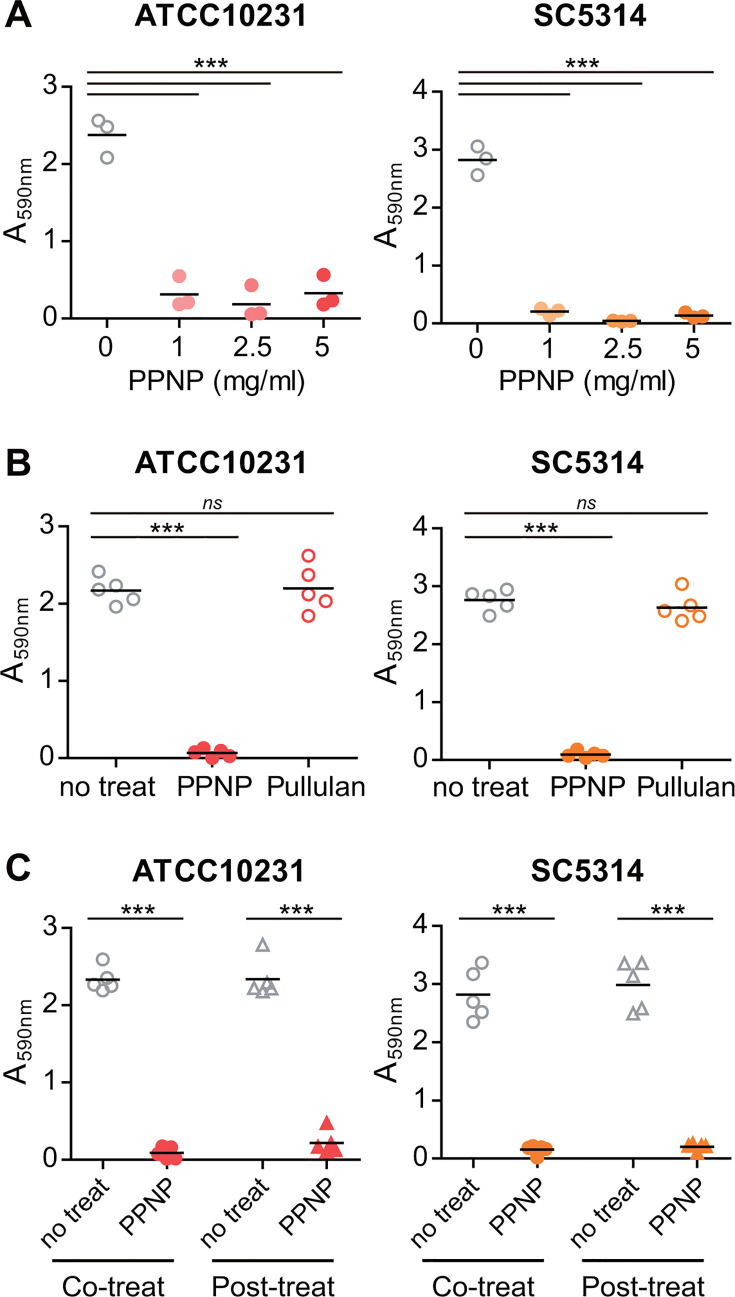
PPNPs inhibit *C. albicans* biofilm formation. (**A**) Inhibitory effect of PPNPs on the biofilm formation of *C. albicans* cells on a plastic surface. ATCC10231 and SC5314 cells were incubated in SC media containing 10% FBS with the indicated concentrations of PPNPs at 37°C for 48 h. (**B**) NP-specific inhibitory effect of PPNPs on the biofilm formation of *C. albicans* on a plastic surface. ATCC10231 cells and SC5314 cells were incubated in SC media containing 10% FBS with 5 mg/mL of PPNPs or pullulan at 37°C for 48 h. (**C**) Inhibitory effect of PPNPs on the maturation of intermediate biofilm of *C. albicans*. ATCC10231 cells and SC5314 cells were incubated in SC media containing 10% FBS. For co-treatment conditions, cells were exposed to a 48-h incubation at 37°C, with or without the presence of 5 mg/mL PPNPs. Alternatively, post-treatment conditions involved a 90-min incubation without PPNPs, followed by supplementation with 5 mg/mL PPNPs for an additional duration of 46 h and 30 min. Biofilm was quantified by crystal violet staining. Asterisks indicate significant differences (paired two-tailed Student’s *t*-test): ***P < 0.001; *ns*, not significant.

Adhesion to abiotic surfaces represents an early stage of biofilm formation in *C. albicans*. Given that PPNPs inhibit the adhesion of *C. albicans* to abiotic surfaces, we examined whether PPNPs also have an inhibitory effect on the biofilm formation of *C. albicans*. While *C. albicans* cells formed dense biofilms after incubation at 37°C for 48 h, their biofilm formation was significantly reduced upon treatment with PPNPs at concentrations above 1 mg/mL (Fig. 4A). In contrast, treatment with pullulan did not exhibit any inhibitory effect on the biofilm formation of *C. albicans* (Fig. 4B).

In general, the biofilm formation process of *C. albicans* is commonly divided into three stages (51, 52). Early biofilm development occurs within the first 90 min after the initial adhesion of *C. albicans* cells to abiotic surfaces. An intermediate biofilm forms by 24 h after the initial attachment, and a mature biofilm develops within 48 h. Next, we investigated whether PPNPs inhibit the maturation of a biofilm that has already begun to develop. *C. albicans* cells were incubated for 90 min, then treated with 5 mg/mL PPNPs (post-treatment), and incubated for a total of 48 h. As shown in Fig. 4C, biofilm development was almost completely inhibited in both *C. albicans* strains, similar to the results when *C. albicans* was treated with PPNPs for 48 h from the beginning (co-treatment). Taken together, these results suggest that PPNPs inhibit the biofilm formation of *C. albicans* and can even hinder biofilm development after the initial stages have been established.

### PPNPs downregulate the expression of hypha-related genes

**Fig 5:**
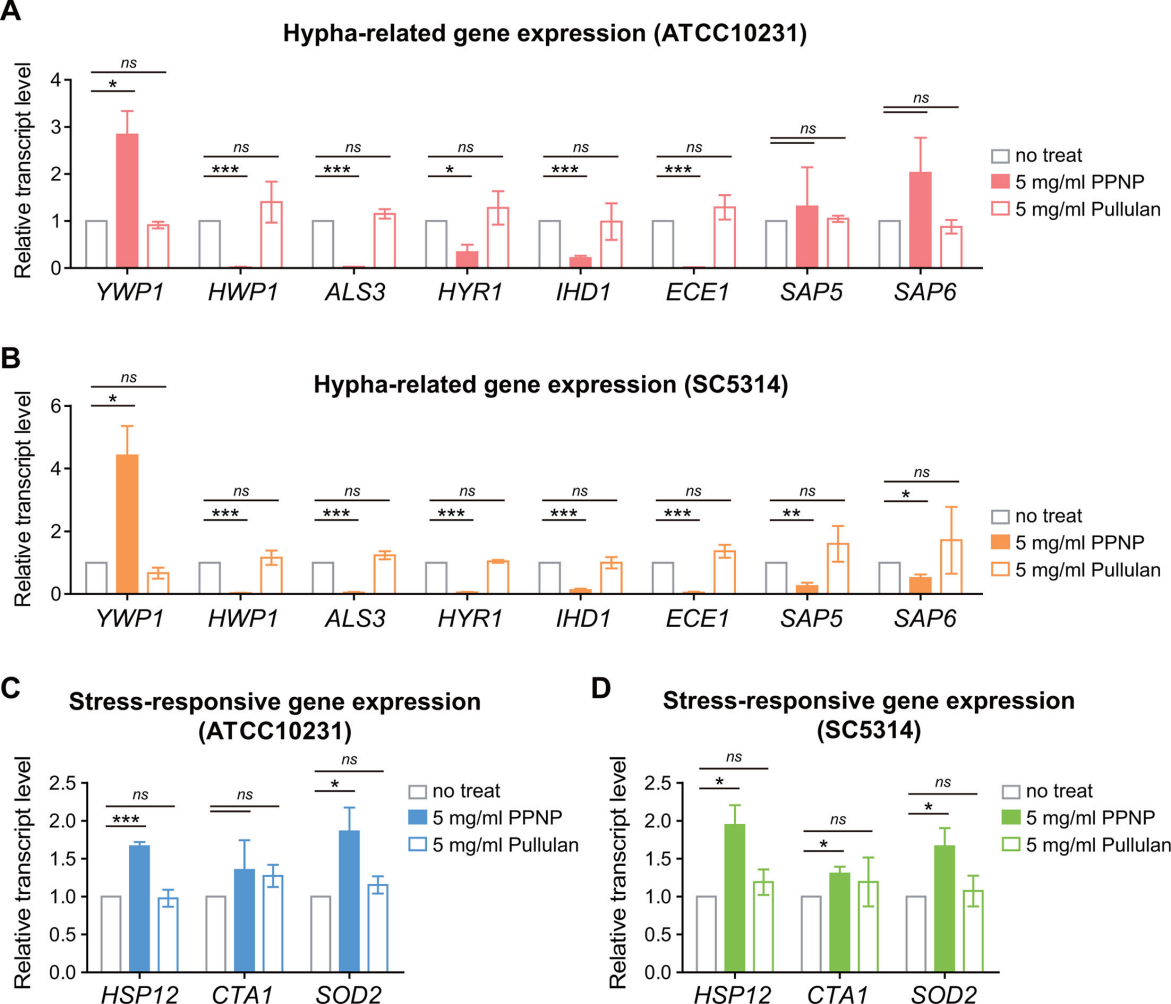
PPNPs modulate *C. albicans* gene expression. (**A**) Downregulation of hypha-specific gene expression by PPNPs in ATCC10231 cells. (**B**) Downregulation of hypha-specific gene expression by PPNPs in SC5314 cells. (**C**) Upregulation of stress-responsive gene expression by PPNPs in ATCC10231 cells. (**D**) Upregulation of stress-responsive gene expression by PPNPs in SC5314 cells. Cells were incubated in SC media containing 10% FBS with 5 mg/mL of PPNPs or pullulan at 37°C for 1 h. Total RNA was extracted, and the transcript level was measured by qRT-PCR. Amplification efficiencies were validated and normalized against *GPD1*. Asterisks indicate significant differences (paired two-tailed Student’s *t*-test): *P < 0.05; **P < 0.01; ***P < 0.001; *ns*, not significant.

The yeast-to-hypha transition of *C. albicans* is crucial for its pathogenicity (13, 14). This morphological transition is regulated by the expression of hypha-related genes, which are induced by various signal transduction pathways (12, 53, 54). Representative hypha-related genes include *HWP1* and *ALS3*, which encode cell wall proteins involved in surface adhesion; *SAPs*, which encode proteases; and *ECE1*, which encodes a cytolytic toxin (55). Because PPNPs did not affect the growth of *C. albicans* even at a concentration of 5 mg/mL and only inhibited its hyphal growth and adhesion to abiotic surfaces, we hypothesized that PPNPs might regulate the expression of hypha-related genes. To test this, we first examined the expression of *HWP1* and *YWP1*, which encode cell wall protein expressed in the hypha and yeast forms, respectively, using quantitative reverse transcription-PCR (qRT-PCR). Notably, when *C. albicans* cells were treated with 5 mg/mL of PPNPs, the expression of *YWP1* increased while the expression of *HWP1* decreased, compared to untreated cells (Fig. 5A and B). This result indicates that cells treated with PPNPs express the yeast form of cell wall protein rather than the hypha form. Furthermore, treatment with 5 mg/mL PPNPs also decreased the expression of *ALS3*, *HYR1*, and *IHD1*, which encode adhesion-related genes, and *ECE1*, which encodes candidalysin. In SC5314 cells, the expression of the *SAP* genes, along with the aforementioned genes, was decreased (Fig. 5B). This modulation in gene expression was not observed when *C. albicans* cells were treated with 5 mg/mL of pullulan. These findings suggest that PPNPs inhibit the adhesion and biofilm formation of *C. albicans* by regulating the expression of hypha-related genes.

It has been previously reported that polysaccharide-based NPs delivered to bacterial cells can increase the expression of heat shock proteins (HSPs) by inducing a mild stress response (43, 44). Given this, we examined whether PPNPs affect various stress responses in *C. albicans*. Interestingly, the expression of *HSP12*, which encodes a small HSP, was increased when treated with 5 mg/mL PPNPs (Fig. 5C and D). Additionally, the expression of other stress-responsive genes, *CTA1* and *SOD2,* which encode catalase and superoxide dismutase, respectively, was also increased. This upregulation of gene expression was not observed when *C. albicans* cells were treated with pullulan. These observations suggest that PPNPs act as a mild stressor in *C. albicans*, inducing the expression of stress-responsive genes.

Taken together, these results suggest that PPNPs decrease the expression of hypha-related genes while increasing the expression of stress-responsive genes. It is likely that the inhibition of the yeast-to-hypha transition mediated by PPNPs, through changes in gene expression, reduces the adhesion of *C. albicans* to abiotic surfaces and the subsequent biofilm formation.

### The Ras/cAMP/PKA signaling pathway is involved in the transcriptional regulation induced by PPNPs

**Fig 6:**
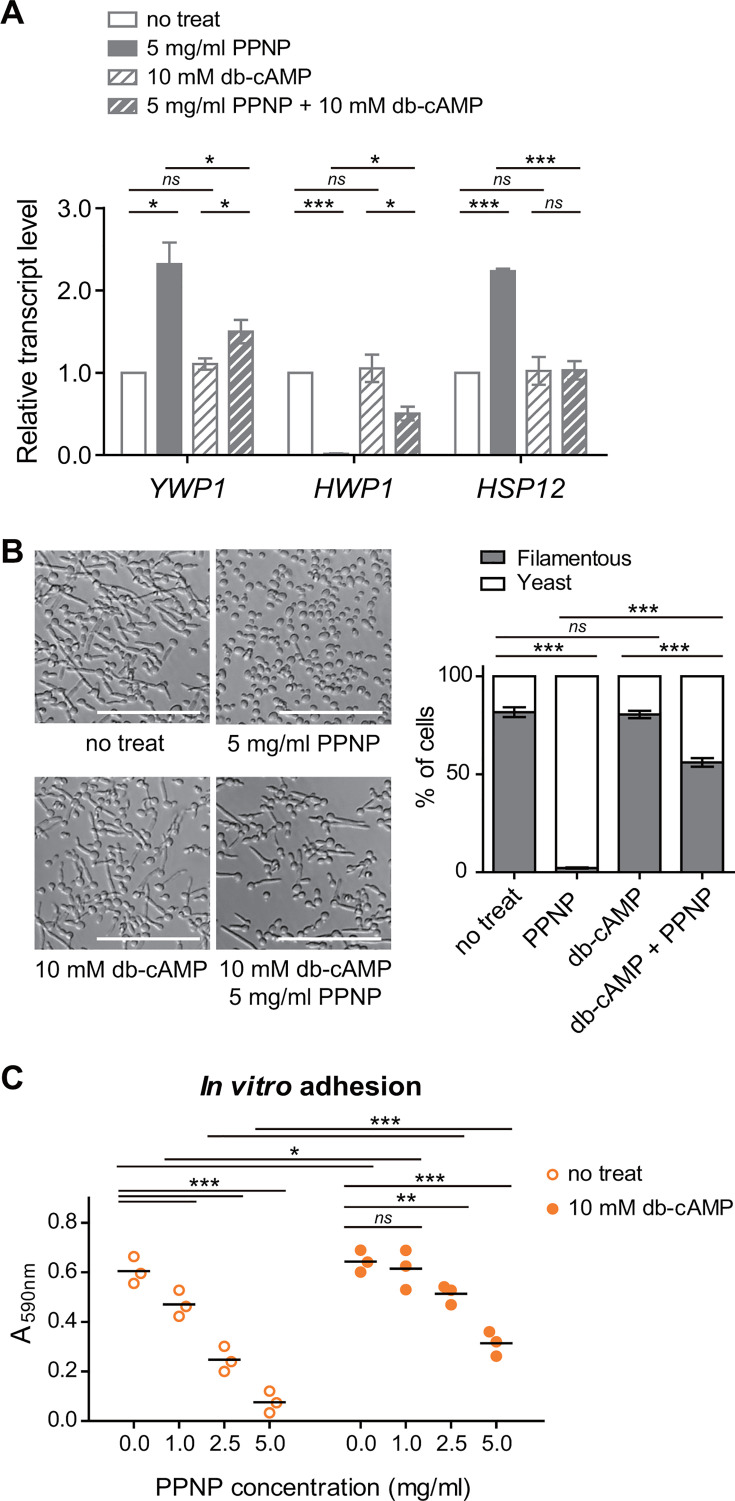
Supplementation with db-cAMP reduces the bioactivity of PPNPs against *C. albicans* cells. (**A**) Transcriptional regulation induced by 5 mg/mL PPNPs and 10 mM db-cAMP. Total RNA was extracted, and transcript levels were measured by qRT-PCR. Amplification efficiencies were validated and normalized against *GPD1*. (**B**) Morphology of *C. albicans* SC5314 cells observed by DIC microscopy. The calculated proportion of yeast cells and filamentous cells is shown on the right. (**C**) *In vitro* adhesion of *C. albicans* cells quantified by crystal violet staining. *C. albicans* SC5314 cells were incubated in SC media containing 10% FBS with the indicated concentrations of PPNPs and 10 mM db-cAMP at 37°C for 1 h (**A and C**) or 2 h (**B**). Asterisks indicate significant differences (paired two-tailed Student’s *t*-test): *P < 0.05; **P < 0.01; ***P < 0.001; *ns*, not significant.

Several signaling pathways regulate hypha-related genes, with the Ras/cAMP/PKA signaling pathway standing out. This pathway is known for promoting the expression of hypha-related genes, while its downregulation triggers the expression of stress-responsive genes (53, 56). Given our observation of increased expression of stress-responsive genes upon treatment with PPNPs, it is plausible that PPNPs induce a mild stress response in *C. albicans*, leading to the downregulation of Ras/cAMP/PKA signaling and the subsequent suppression of hypha-related gene expression. Several molecules that suppress the pathogenicity of *C. albicans* are known to downregulate the Ras/cAMP/PKA signaling pathway, and the inhibitory effects of these molecules can often be reversed by the external supplementation of cell-permeable dibutyl-cAMP (db-cAMP) (57–60). To test our hypothesis, we examined the transcriptional regulation by PPNPs in *C. albicans* SC5314 cells with exogenous supplementation of db-cAMP. As shown in Fig. 6A, the expression of *YWP1*, which increased with 5 mg/mL of PPNP treatment, significantly decreased upon treatment with 10 mM db-cAMP. Similarly, the expression of *HWP1*, which was reduced by 5 mg/mL PPNP treatment, showed a lesser reduction when treated with 10 mM db-cAMP. On the other hand, the expression of the stress-responsive gene *HSP12*, which was upregulated by 5 mg/mL PPNPs, did not increase when 10 mM db-cAMP was supplemented. These results suggest that the upregulation of stress-responsive genes by PPNPs is dependent on the Ras/cAMP/PKA signaling pathway, while the downregulation of hypha-related genes is partially dependent on this pathway.

Next, we investigated the effects of db-cAMP supplementation on the *in vitro* adhesion and hyphal growth of *C. albicans* SC5314 cells. Consistent with the transcript data, hyphal growth, which was significantly reduced to about 2% with 5 mg/mL PPNP treatment, increased to 56% when 10 mM db-cAMP was added (Fig. 6B). Additionally, the suppression of *in vitro* adhesion was reduced when 5 mg/mL of PPNPs and 10 mM of db-cAMP were administered together, compared to PPNPs alone (Fig. 6C). Taken together, these results suggest that the stress response induced by 5 mg/mL PPNPs is mediated by the Ras/cAMP/PKA signaling pathway. Moreover, they indicate that the downregulation of the Ras/cAMP/PKA signaling pathway contributes to the decreased expression of hypha-related genes, leading to the inhibition of hyphal growth and *in vitro* adhesion following PPNP treatment.

### PPNPs inhibit the adhesion of *C. albicans* cells to human epithelial cells

**Fig 7:**
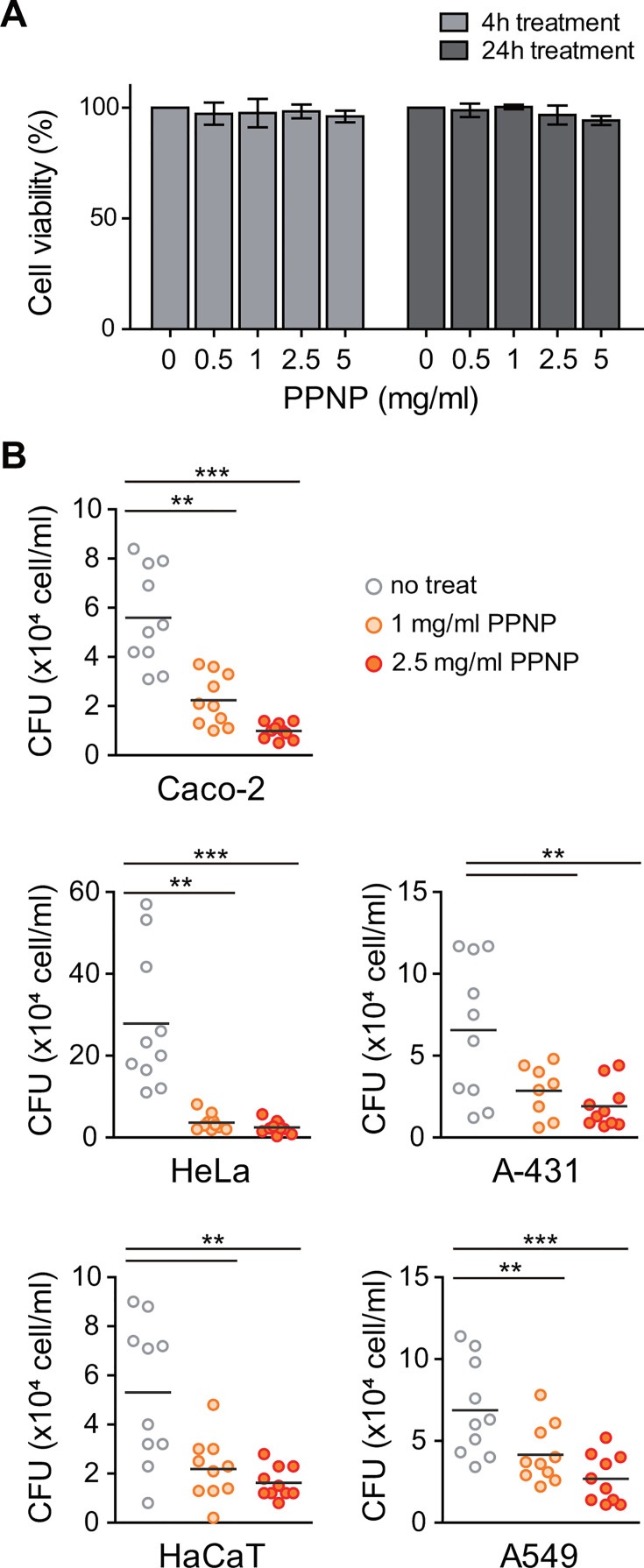
PPNPs inhibit *C. albicans* adhesion to human epithelial cells. (**A**) Cytotoxicity test of PPNPs on human cells. HEK293A cells were cultured in DMEM media containing 10% FBS. Indicated concentrations of PPNPs were added and incubated at 37°C. Cell viability was examined by CCK-8 cell viability assay. (**B**) Inhibitory effect of PPNPs on the adhesion of *C. albicans* to human epithelial cells. *C. albicans* cell suspension with or without PPNPs was added to the monolayer cultures of indicated cell lines and was incubated at 37°C for 3 h. Adhered cells were collected using trypsin-EDTA, and CFUs were counted. Asterisks indicate significant differences (paired two-tailed Student’s *t*-test): **P < 0.01; ***P < 0.001.

Building upon our findings that highlighted PPNPs’ inhibition of *C. albicans* attachment to abiotic surfaces, we investigated their potential to impede the adhesion of *C. albicans* to human epithelial cells. First, we examined the effect of PPNPs on the viability of human cells using a CCK-8 cell viability assay. Exposure to various concentrations (0.5 to 5 mg/mL) of PPNPs for 4 or 24 h did not adversely affect the viability of HEK293A cells (Fig. 7A), indicating that PPNPs up to 5 mg/mL are non-toxic to human cells. Subsequently, to assess the adhesion of *C. albicans* to human epithelial cells, we conducted a cell adhesion assay using Caco-2 cells derived from human colon adenocarcinoma (61). In this assay, *C. albicans* SC5314 cells were inoculated onto the Caco-2 monolayer culture and incubated for 3 h. As shown in Fig. 7B, adhesive *C. albicans* cells were efficiently detected on the Caco-2 monolayer. In contrast, when *C. albicans* was inoculated onto the Caco-2 monolayer culture treated with 1 or 2.5 mg/mL of PPNPs, we observed a substantial reduction in the number of attached *C. albicans* cells compared to the untreated condition. Next, we tested whether PPNPs also inhibit *C. albicans* attachment to other human epithelial cells commonly affected by candidiasis, including HeLa (cervix), A-431 (vulvovaginal tissue), HaCaT (skin), and A549 (lung) cells. Notably, treatment with 1 or 2.5 mg/mL of PPNPs significantly reduced *C. albicans* adhesion to all these cell lines (Fig. 7B). Collectively, these findings underscore the efficacy of PPNPs in preventing *C. albicans* attachment to cultured human epithelial cells. Moreover, given the non-toxic nature of PPNPs (up to 5 mg/mL) toward human cells, these results suggest the potential of PPNPs as anti-adhesion agents against *C. albicans* on human cells.

### PPNPs inhibit the *in vivo* pathogenicity of *C. albicans* against *C. elegans*

**Fig 8:**
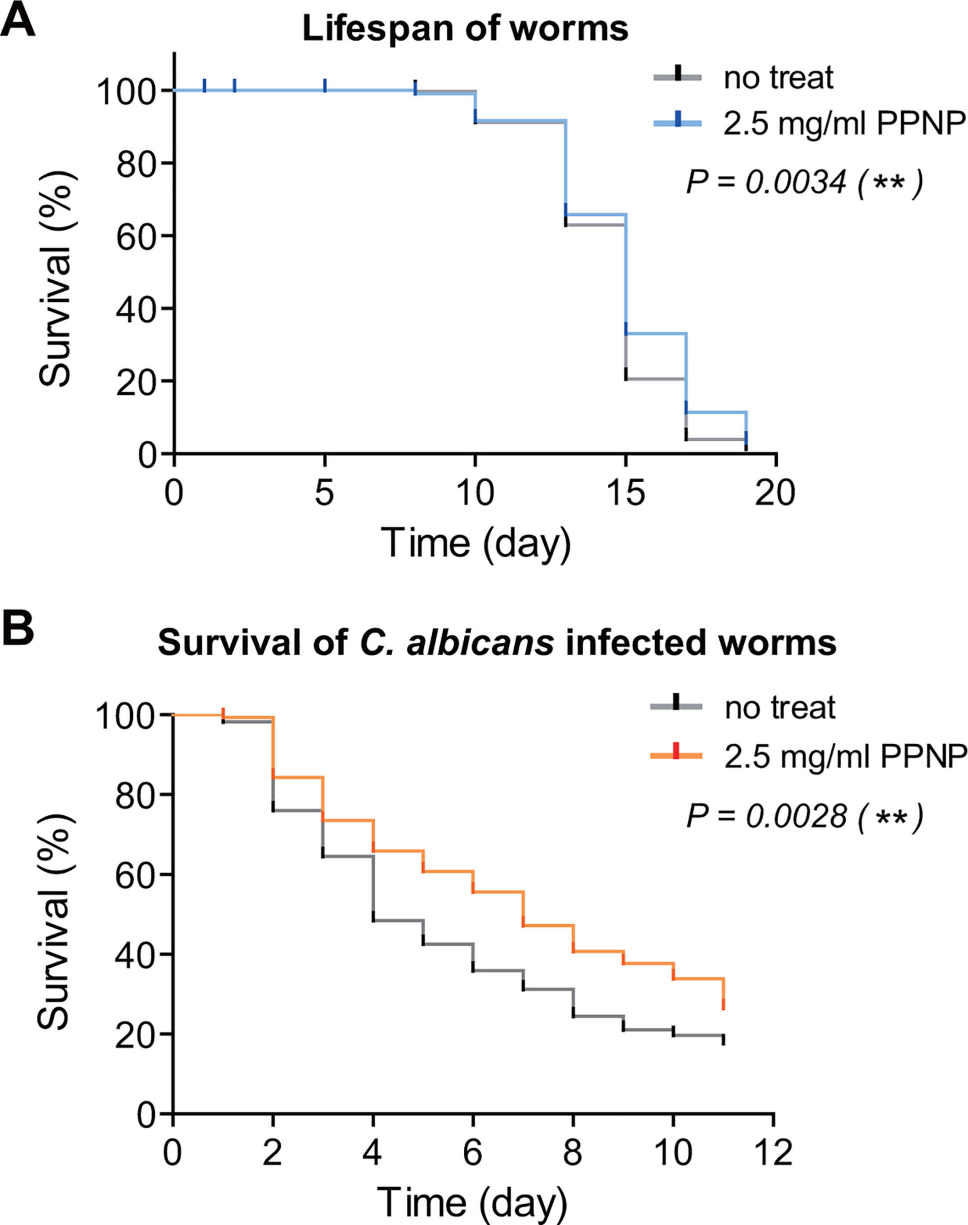
PPNPs inhibit the *in vivo* pathogenicity of *C. albicans* in *C. elegans*. (**A**) Non-toxic nature of PPNPs against nematodes. *C. elegans* N2 worms were fed with *E. coli* OP50 as the standard diet in the absence or presence of 2.5 mg/mL of PPNPs. (**B**) Antagonistic effect of PPNPs on the *in vivo* pathogenicity of *C. albicans* in nematodes. *C. elegans* N2 worms were fed with *C. albicans* SC5314 cells with or without 2.5 mg/mL PPNPs at 30°C for 4 h, and then transferred to pathogen-free liquid medium. Dead worms were counted, and live worms were transferred to a fresh liquid medium. *C. elegans* lifespan and survival were examined using the Kaplan-Meier method. Asterisks indicate significant differences (log-rank test): **P < 0.01.

Encouraged by the results showing that PPNPs protect human epithelial cells from *C. albicans* adhesion, we investigated their potential to reduce the pathogenicity of *C. albicans* in a live animal model. We chose the nematode *C. elegans*, which is widely used to study the virulence of diverse pathogenic microorganisms, including *Candida* species (62–64). First, we examined the impact of PPNPs on the lifespan of *C. elegans*. When *C. elegans* was fed with *Escherichia coli* OP50 as the standard diet, the age of 50% mortality was day 15, regardless of the presence of 2.5 mg/mL PPNPs (Fig. 8A). Although the age of 90% mortality was day 17 in the absence of PPNPs, it was day 19 in the presence of 2.5 mg/mL PPNPs. This observation underscores the non-toxic nature of 2.5 mg/mL PPNPs toward nematode growth. Next, we exposed nematodes to *C. albicans* SC5314 cells for 4 h to induce *Candida* infection, then monitored their survival rate. Nematodes infected with *Candida* exhibited a rapid decline in survival, with 50% succumbing by day 4 (Fig. 8B). Remarkably, when nematodes were exposed to *C. albicans* in the presence of 2.5 mg/mL PPNPs, their mortality was significantly reduced; 50% of the *C. albicans*-infected worms survived until day 7. These results suggest that PPNPs mitigate the mortality rate by suppressing *C. albicans* pathogenicity in living organisms.

## DISCUSSION

Despite the common occurrence of superficial or systemic candidiasis, the limited availability of antifungal agents for its treatment has generated considerable interest in alternative therapeutic approaches. Alternative strategies, such as the utilization of nanomaterials for efficient delivery of antifungal agents (65–69) and the exploration of other microorganisms or small molecules with fungicidal or fungistatic properties (70–72), are gaining attention. Although numerous studies have investigated the potential of NPs in combating pathogenic microbes such as *C. albicans*, most of these investigations have primarily focused on the physical properties of NPs related to their ability to infiltrate the extracellular matrix, penetrate biofilms, and disrupt cellular integrity via surface attachment (67, 68, 73–75). Consequently, research concerning the bioactivity of NPs themselves remains at an early stage. In this study, we demonstrated that when pullulan, a water-soluble polysaccharide, was transformed into the form of NPs, they effectively inhibited the hyphal growth of *C. albicans* regardless of the strain. We also validated through *in vitro* adhesion assays that PPNPs exert an inhibitory effect on the adhesion and subsequent biofilm formation of *C. albicans* on abiotic surfaces. Notably, even at a concentration of 1 mg/mL, PPNPs were highly effective and nearly completely inhibited biofilm formation, highlighting their potential to impede biofilm maturation.

Notably, our data indicate that the inhibitory effects of PPNPs on the *in vitro* adhesion of *C. albicans* are not related to the presence of pullulan, raising the possibility that polymeric NPs other than PPNPs may have similar effects. To test this possibility, we investigated whether other types of NPs could also hinder the adhesion of *C. albicans* to abiotic surfaces. Similar to PPNPs, we synthesized acetyl pullulan NPs (APNPs) using the acetyl group instead of the phthalic group (Fig. S3A) and phthalic-resistant starch NPs (PSNPs) using resistant starch, which resists digestion in the small intestine, instead of pullulan (Fig. S3B). An *in vitro* adhesion assay revealed that not only PPNPs but also APNPs and PSNPs inhibited the attachment of *C. albicans* to plastic surfaces (Fig. S3C). These results suggest that NPs derived from various polysaccharides can also inhibit the pathogenicity of *C. albicans*.

We found that PPNPs regulate the pathogenicity of *C. albicans* at the gene expression level. PPNPs induced the upregulation of stress-responsive genes and inhibited hyphal growth, *in vitro* adhesion, and biofilm formation through the downregulation of hypha-related genes. Results from the exogenous db-cAMP supplementation experiment confirmed that the upregulation of stress-responsive genes is dependent on the Ras/cAMP/PKA pathway (Fig. 6). However, the downregulation of hypha-related genes suggests the involvement of additional pathways beyond the Ras/cAMP/PKA pathway. To explore other potential regulatory pathways, we performed a transcriptome analysis of *C. albicans* SC5314 cells in response to treatment with 5 mg/mL of PPNPs. Hierarchical clustering of transcriptomes between the PPNP-treated group and the untreated control group revealed that each condition has a distinct transcriptome profile (Fig. S4A). When considering genes with a statistically significant change in expression greater than twofold (Benjamini-Hochberg adjusted *P* value < 0.05) as differentially expressed genes, only a small number of genes were differentially expressed following PPNP treatment (62 downregulated genes and 28 upregulated genes) (Fig. S4B). As anticipated from the qRT-PCR data, most of the genes downregulated by PPNP treatment were associated with the fungal-type cell wall, biofilm formation, and interactions with other species (Fig. S5). Conversely, among the upregulated genes, several were involved in the utilization of metal ions, particularly iron ions, including *FET99, FET31, FRE10, CLF2, CLF4, PGA7,* and *RBT5* (Fig. S6). Because the utilization of metal ions is crucial for the pathogenic expression of *C. albicans* (76), these findings suggest that negatively charged PPNPs may inhibit hypha-related gene expression by disrupting iron ion utilization in *C. albicans* cells. To further investigate this, we examined the effect of iron ion supplementation on the suppression of hypha-related genes by PPNPs. Supplementation with 100 µM Fe²^+^ and Fe³^+^ reduced the inhibition of hypha-related gene expression induced by 5 mg/mL PPNPs (Fig. S7). However, iron ion supplementation did not affect the expression of stress-responsive genes induced by PPNPs. These results suggest that iron ion metabolism, in addition to the Ras/cAMP/PKA signaling pathway, may contribute to the inhibition of *C. albicans* pathogenicity by PPNPs. Further investigation is needed to elucidate the effects of PPNPs on the utilization of metal ions in *C. albicans*.

Polymeric NPs are widely used to deliver bioactive molecules, such as nucleic acids and drugs, into cells and are known to enter mammalian cells via endocytosis (39, 40). To investigate whether polymeric NPs could be internalized into fungal cells, we examined *C. albicans* cells treated with fluorescein isothiocyanate (FITC)-conjugated PPNPs using fluorescence microscopy. We observed that FITC-PPNPs localized within membranous structures stained with FM4-64, specifically endosomes and vacuoles (Fig. S8A) (77). In contrast, the accumulation of intracellular FITC-PPNPs was significantly reduced in the endocytosis-defective *end3*Δ/Δ and *snf7*Δ/Δ mutants (Fig. S8B and C) (78, 79). However, despite this reduced internalization, treatment with PPNPs still resulted in the inhibition of *in vitro* adhesion, downregulation of hypha-related genes, and upregulation of stress-responsive genes in the *end3*Δ/Δ and *snf7*Δ/Δ mutants (Fig. S9). These results suggest that although PPNPs enter *C. albicans* cells via endocytosis, their inhibitory effects on *C. albicans* are independent of this process.

The formation of *C. albicans* biofilms poses a significant challenge in preventing candidiasis. The extracellular matrix, secreted by *Candida* cells during biofilm formation, acts as a barrier that hinders the penetration of antifungal agents, making the complete eradication of *Candida* colonies difficult (16, 18). *Candida* cells thriving within these protective biofilms can continue to disperse, leading to infections, particularly in immunocompromised patients. Furthermore, the exceptional ability of *C. albicans* to form biofilms facilitates the formation of mixed-species biofilms with other microorganisms, exacerbating the risk. For example, *Streptococcus mutans*, an oral bacterium, adheres to *C. albicans* biofilms in the oral cavity, spreads rapidly along the biofilm, and contributes to more extensive tooth decay (80). In addition, other fungi, such as the multidrug-resistant *Candida auris*, which cannot form mature biofilms independently, can cause diseases by colonizing with the assistance of *C. albicans* biofilms (20, 81, 82). Therefore, PPNPs not only show potential in suppressing candidiasis but also hold promise in inhibiting various diseases associated with *C. albicans* biofilms.

Although eradicating *Candida* cells using a high dose of antifungal drugs may seem like the simplest approach to dealing with *Candida* biofilm and candidiasis, it is important to consider that *Candida* species are also members of the normal flora. Eliminating these fungi could disrupt the balance of microorganisms in the normal flora, potentially leading to dysbiosis and harm to host cells (6). Previous studies have shown that suppressing the pathogenicity of *C. albicans* through genetic manipulation increases the survival rate of infected mice (13, 14). Additionally, it has been observed that the non-pathogenic form of *Candida* residing in the intestines of mice reduces mortality from systemic candidiasis caused by intravenous challenge (83). Although PPNPs effectively inhibited *C. albicans* adhesion to abiotic surfaces and biofilm formation, they did not have lethal effects against *C. albicans* (Fig. S2). Therefore, using PPNPs to inhibit pathogenicity without killing *C. albicans* cells may actually be a safer approach to treating candidiasis. Furthermore, when fluconazole, a representative antifungal agent for candidiasis, was administered in combination with PPNPs, the suppression of *C. albicans* survival was more effective compared to treatment with fluconazole alone (Fig. S10). Thus, combining PPNPs, which inhibit the pathogenicity of *C. albicans*, with a lower dose of antifungal agents may offer a safer and more effective strategy than using a high dose of antifungal agents alone.

The effectiveness of PPNPs against superficial candidiasis was validated using an *ex vivo* adhesion assay in which *C. albicans* was exposed to human epithelial cells. PPNPs significantly inhibited the attachment of *C. albicans* to epithelial cells derived from various tissues. Additionally, we confirmed their ability to inhibit the *in vivo* pathogenicity of *C. albicans* against the model organism *C. elegans*. Notably, PPNPs exhibited no detrimental effects on the lifespan of nematodes or the viability of human cell lines. These results underscore the potential of PPNPs as inhibitors of *Candida* infections. Furthermore, a previous study demonstrated the effectiveness of PPNPs in promoting the recovery of dysbiosis in the intestines of a mouse model (46). These findings suggest that PPNPs not only serve as inhibitors of *Candida* infections but also hold promise as nano-prebiotics. Additionally, with their low cost and straightforward synthesis, polysaccharide NPs represent a promising avenue for novel therapeutics against candidiasis.

## MATERIALS AND METHODS

### Chemicals, strains, cell lines, and growth conditions

Pullulan used in this study was purchased from Shandong Freda Biotechnology, and other chemicals were purchased from Sigma-Aldrich. *C. albicans* strain ATCC10231 and SC5314 cells were grown in YPD medium (1% yeast extract, 2% peptone, and 2% glucose) or SC medium (0.67% yeast nitrogen base with appropriate amino acids and 2% glucose) or brain heart infusion (BHI) medium. Unless otherwise stated, yeast cells were pre-incubated in YPD medium at 30°C for 48 h. All broths were purchased from BD Difco. For db-cAMP supplementation, bucladesine sodium salt (HY-B0764, Med Chem Express) was added to media at a final concentration of 10 mM. HEK293A (R70507, Invitrogen), Caco-2 (30037.1, KCLB), HeLa (10002, KCLB), HaCaT (provided by GPCR Therapeutics Inc.), A-431 (provided by GPCR Therapeutics Inc.), and A549 cells (CCL-185, ATCC) were maintained in RPMI 1640 or Dulbecco's modified Eagle medium (DMEM), supplemented with 10% fetal bovine serum (FBS), 100 U/mL penicillin, and 100 µg/mL streptomycin, at 37°C in 5% CO_2_. All cell culture media were purchased from Cytiva HyClone. *C. elegans* Bristol N2 strain was cultured on nematode growth medium (NGM) agar plates (2% agar, 51.3 mM NaCl, 0.25% peptone, 12.93 mM cholesterol, 0.5 mM CaCl_2_, 1 mM MgSO_4_, 20 mM KH_2_PO_4_, 5.167 mM K_2_HPO_4_) overgrown with *E. coli* OP 50 strain.

### Preparation of PPNPs

PPNPs were prepared according to the previous paper (44) with some modifications. Briefly, 1 g of pullulan and 24 mg of dimethylaminopyridine (DMAP) as a catalyst were dissolved in 10 mL of dimethylformamide (DMF). Subsequently, 2.64 g of phthalic anhydride was added on a molar ratio of 9:1 (phthalic anhydride:pullulan), and then the reaction was carried out at 54°C for 48 h under nitrogen bubbling. The obtained PP was dialyzed against DMF to remove unreacted phthalic anhydride and DMAP, and then dialyzed against double-distilled water at room temperature for 48 h. After removing unreacted pullulan by ultracentrifugation, the obtained PP was lyophilized. Finally, the self-assembled PPNPs were obtained by dissolving 100 mg of lyophilized PP in 3 mL of DMF and dialysis in appropriate medium or triple-distilled water at 4°C for 48 h.

### Characterization of PPNPs

Phthalyl group contents in PPNPs were measured by 600 MHz ^1^H-NMR spectroscopy (AVANCE 600, Bruker). For NMR measurements, lyophilized PPNPs were dissolved in dimethyl sulfoxide (DMSO)-d6. The surface morphology of PPNPs was visualized using FE-SEM (SIGMA, Carl Zeiss). For FE-SEM, lyophilized PPNPs were mounted onto stubs with adhesive copper tape and coated with gold under a vacuum using a coating chamber (Ion Sputter Coater G-20, GSEM). The coated samples were observed and imaged with FE-SEM. The size and zeta potential of PPNPs were measured by a DLS spectrophotometer (DLS-7000, Otsuka Electronics) and an ELS spectrophotometer (ELS Z-1000, Otsuka Electronics). For DLS and ELS measurements, PPNPs were dispersed in triple-distilled water.

### Examination of hyphal growth

Forty-eight-hour-old *C. albicans* ATCC10231 cells and SC314 cells were harvested, washed with phosphate-buffered saline (PBS), and resuspended in fresh SC medium supplemented with 10% FBS and appropriate concentrations of PPNPs or pullulan and then incubated at 37°C for 2 h. After incubation, cells were washed with PBS containing 20 mM NaN_3_ and were observed using a differential interference contrast (DIC) microscopy (Nikon Eclipse E1 microscope with a Plan Fluor 100×/1.30 NA oil immersion objective, Nikon). Assays were conducted three times.

### *In vitro* adhesion and biofilm formation assay

*In vitro* adhesion and biofilm formation of *C. albicans* strains ATCC10231 and SC5314 were monitored using the method previously described (72), with some modifications. *C. albicans* cells were harvested, washed with PBS, and resuspended in fresh SC medium supplemented with 20% FBS to achieve a final concentration of 5 × 10^6^ cell/mL. Fifty microliters of the cell suspension and 50 µL of SC medium containing PPNPs or pullulan were added to each well of a 96-well cell culture plate. To monitor adhesion, the plates were incubated at 37°C for 1 h with mild shaking (90 rpm). For biofilm formation assessment, the plates were incubated for 48 h. To monitor intermediate biofilm maturation, 50 µL of the cell suspension was incubated for 90 min. Subsequently, 50 µL of SC medium with or without 5 mg/mL PPNPs was added to each well, followed by further incubation for 46 h and 30 min. Adherent cells were observed using DIC microscopy (Nikon Eclipse E1 microscope with a Plan Fluor 100×/1.30 NA oil immersion objective, Nikon), and the absorbance of crystal violet-stained cells was measured at 590 nm using a microplate reader (FlexStation 3 Multi-Mode Microplate Reader, Molecular Devices). Assays were conducted at least three times with six replicates.

### Quantification of transcript levels

*C. albicans* was harvested and resuspended in fresh SC medium supplemented with 10% FBS containing 5 mg/mL of PPNPs or pullulan to achieve a final concentration of 5 × 10^6^ cell/mL. After incubation at 37°C for 2 h, total RNA was extracted from the cells using the hot PCA extraction with SDS method (84). cDNA was synthesized using the ReverTra Ace qPCR RT Kit (TOYOBO, FSQ-101). The amount of each transcript was analyzed by quantitative real-time reverse transcription-PCR (QuantStudio 3 Real-Time PCR System, Applied Biosystems) and the 2X Real-Time PCR Master Mix (BioFACT, DQ362-40h). Primers used for the amplification of each transcript are listed in Table S1. The transcript level of each gene was measured, normalized against that of *GPD1*, and quantified using the 2^-ΔΔC^_T_ method (85). All relative transcript levels were determined from three independent experiments with duplicates.

### Cytotoxicity assay

The cytotoxicity of PPNPs on human cells was determined using the CCK-8 assay (86). PPNPs were prepared in DMEM by dialyzing DMF-dissolved PP in DMEM at 4°C for 48 h. HEK293A cells were seeded into 96-well plates at a density of 2 × 10^4^ cells/well and grown for 1 d. Cells were then treated with PPNPs-containing media for either 4 or 24 h. Following treatment, 10 µL of CCK-8 solution (DOJINDO, CK04) was added to each well and incubated for 4 h. The absorbance was measured at 450 nm using a microplate reader (EnVision Multilabel Plate Readers, PerkinElmer). Assays were performed three times with six replicates.

### *Ex vivo* adhesion assay

Caco-2, HeLa, A-431, HaCaT, and A549 cells were utilized to monitor the adhesion of *C. albicans* SC5314 cells to human epithelial cells. To establish monolayers, Caco-2 cells were seeded into 24-well plates at a density of 4 × 10^5^ cells/well and cultured for 7 d, while HeLa, A-431, HaCaT, and A549 cells were seeded at a density of 1 × 10^5^ cells/well and grown for 2–3 d. The monolayers were then exposed to 2 × 10^6^ cells/mL of *C. albicans* for 3 h. Non-adherent cells were subsequently removed by washing three times with Dulbecco's phosphate-buffered saline (DPBS). Adherent cells were harvested by treating with trypsin-EDTA (0.25% trypsin and 0.02% EDTA) for 10 min at 37°C, followed by resuspension in 500 µL of DPBS. Enumeration of *C. albicans* colony forming units (CFUs) was performed on YPD plates. Assays were conducted 10 times with duplicates.

### *C. elegans* lifespan assay

To assess the toxicity of PPNPs on *C. elegans*, eggs were collected from N2 worms at day 1 of adulthood grown on *E. coli* OP50 on NGM plates with or without 2.5 mg/mL of PPNPs. The progeny were cultured at 20°C for 2 d, and L4 stage worms were then transferred to NGM plates containing 50 µM of 5-fluorodeoxyuridine (FUDR) for lifespan measurements. Approximately 30 *C*. *elegans* animals at the L4 stage were maintained on *E. coli* on FUDR plates with or without 2.5 mg/mL of PPNPs at 20°C, and the number of live and dead animals was recorded every 2 or 3 d. Worms that exhibited no movement upon gentle touch were considered dead. Lifespan data were obtained from three independent experiments, each comprising approximately 100 worms.

### *C. elegans* infection assay

The *C. elegans–C. albicans* infection assay was performed as previously described (62) with some modifications. To prepare the *C. albicans* infection plates, the SC5314 strain was grown overnight in YPD media at 30°C. A mixture of 75 µL of yeast culture and 75 µL of distilled water, with or without 5 mg/mL of PPNPs, was then incubated overnight at 30°C on BHI agar plates containing 45 µg/mL of kanamycin. Fifty hermaphrodite progeny animals at the L4 stage were transferred to the infection plates and incubated at 30°C for 4 h. The *C. elegans* was subsequently washed three times with M9 buffer (22.04 mM KH_2_PO4, 42.27 mM Na_2_HPO_4_, 85.56 mM NaCl, 1 mM MgSO_4_). Thirty worms were then transferred to a 6-well cell culture plate containing liquid medium (80% BHI, 20% M9 buffer) and were incubated at 30°C. Live and dead animals were counted daily for 11 d, with live worms transferred to a fresh medium at each count. Survival was determined from five independent experiments, each comprising approximately 30 worms.

### Statistical analysis

Differences in the ratios of filamentous cells, *in vitro* and *ex vivo* adhesion, *in vitro* biofilm formation, transcript levels of *C. albicans,* and the cytotoxicity of PPNPs were determined by paired two-tailed Student’s *t*-test using GraphPad Prism (version 5.01). The lifespan and survival of *C. elegans* were examined using the Kaplan-Meier method, and differences were determined with the log-rank test (OASIS 2; http://sbi.postech.ac.kr/oasis2).

## SUPPLEMENTAL MATERIAL

Supplemental material spectrum.01048-24-s0001.pdf Table S1; Fig. S1 to S10.

ASM does not own the copyrights to Supplemental Material that may be linked to, or accessed through, an article. The authors have granted ASM a non-exclusive, world-wide license to publish the Supplemental Material files. Please contact the corresponding author directly for reuse.
