# Supplementary material for: Pullulan nanoparticles inhibit the pathogenicity of Candida albicans by regulating hypha-related gene expression
Source: Microbiol Spectr. 2024 Nov 14;12(12):e01048-24. doi: 10.1128/spectrum.01048-24 (PMC11619324; doi:10.1128/spectrum.01048-24)
Supplement: Supplemental material — Table S1; Fig. S1 to S10. [file spectrum.01048-24-s0001.pdf]

Supplementary information

Pullulan nanoparticles inhibit the pathogenicity of *Candida albicans* by regulating hypha-related gene expression

Sujin Hong[a,b], Seo-Kyung Kim[c], Christine H. Chung[d,e], Cheol-Heui Yun[c,f], Junho Lee[a,d], Chong-Su Cho[f*], Won-Ki Huh[a,b]**

[a] School of Biological Sciences, Seoul National University, Seoul 08826, Republic of Korea.

[b] Institute of Microbiology, Seoul National University, Seoul 08826, Republic of Korea

[c] Department of Agricultural Biotechnology, Seoul National University, Seoul 08826, Republic of Korea.

[d] Institute of Molecular Biology and Genetics, Seoul National University, Seoul 08826, Republic of Korea

[e] Interdisciplinary Graduate Program in Genetic Engineering, Seoul National University, Seoul 08826, Republic of Korea

[f] Research Institute of Agriculture and Life Sciences, Seoul National University, Seoul 08826, Republic of Korea.

* Corresponding author. Mailing address: Research Institute of Agriculture and Life Sciences, Seoul National University, Seoul 08826, Republic of Korea.

** Corresponding author. Mailing address: School of Biological Sciences, Seoul National University, Seoul 08826, Republic of Korea.

E-mail addresses: chocs@snu.ac.kr (C.-S. Cho), wkh@snu.ac.kr (W.-K. Huh).

Table of Contents

Supplementary materials and methods

Table S1. Oligonucleotides used in this study.

Figure S1. Characteristics of phthalic pullulan nanoparticles (PPNPs).

Figure S2. The effect of PPNPs on the growth of *C. albicans.*

Figure S3. Inhibitory effect of various NPs on *C. albicans* adhesion to abiotic surfaces.

Figure S4. Global transcriptomic change of the *C. albicans* SC5314 response to treatment with 5 mg/ml PPNPs.

Figure S5. Downregulated genes and overrepresented GO term for biological processes downregulated in response to treatment with 5 mg/ml PPNPs.

Figure S6. Upregulated genes and overrepresented GO term for biological processes downregulated in response to treatment with 5 mg/ml PPNPs.

Figure S7. Supplementation of iron ion reduces the inhibitory effect of PPNPs on hypha-related gene expression of *C. albicans* cells.

Figure S8. PPNPs are internalized into *C. albicans* cells through endocytosis.

Figure S9. Inhibitory effect of PPNPs against *C. albicans* cells are independent of their endocytosis.

Figure 10. PPNPs increase the susceptibility of *C. albicans* to fluconazole.

Supplementary materials and methods

Plasmid, chemical and growth condition.

The plasmid for *C. albicans* mutagenesis (pV1524) was a gift from Gerald Fink (Addgene plasmid # 111431; http://n2t.net/addgene:111431; RRID: Addgene_111431). Nourseothricin sulfate was purchased from Med Chem Express (HY-129065), and fluconazole was purchased from Sigma-Aldrich (PHR1160-1G). For iron ion supplementation, $FeSO_4$ and $FeCl_3$ were added to the media at a final concentration of 100 μM each.

Transcriptome analysis

C. albicans SC5314 cells were harvested and resuspended in fresh SC medium supplemented with 10% FBS and 5 mg/ml of PPNPs to achieve a final concentration of 5×10^6 cells/ml. After incubation at 37 ℃ for 2 h, total RNA was extracted from the cells using the hot PCA extraction with SDS method (1). RNA sequencing was performed at Macrogen (Seoul, Korea). Briefly, cDNA libraries for RNA-seq analysis were prepared from total RNA samples using an Illumina TruSeq Stranded mRNA Library Prep Kit. RNA sequencing was performed using an Illumina NovaSeq 6000 platform (San Diego, CA) to obtain 100-bp paired-end reads.

For the RNA-seq data analysis, the paired-end reads were aligned to the *C. albicans* strain SC5314 reference genome (Assembly 21) (http://www.candidagenome.org/) using STAR (Version 2.7.10b) (2). Using the iDEP web tool with all parameters set to default (3), read counts were normalized, and differentially expressed genes were analyzed. Specifically, genes with a false discovery rate (FDR)-adjusted *P*-value of less than 0.05 and expression fold changes greater than 2 were considered differentially expressed. Functional enrichment analysis was then performed on these differentially expressed genes to identify overrepresented Gene Ontology (GO) biological processes using the PANTHER classification system (4-6), with an FDR cutoff of 0.05.

C. albicans mutagenesis

*C. albicans end3*Δ/Δ and *snf7*Δ/Δ strains were generated from the *C. albicans* SC5314 strain by gene disruption using a single-plasmid CRISPR/Cas9 system (pV1524) (7, 8). Briefly, for each gene disruption, single guide RNA (sgRNA) was designed using CHOPCHOP Version 3.0 (https://chopchop.cbu.uib.no/) (9). An annealed pair of sg-oligonucleotides was integrated into the BsmBI-digested pV1524 parent vector. Donor DNA, a repair template for homologous recombination, was prepared using primers that induce a deletion of approximately 700 to 1000 bp while including a stop codon. SC5314 cells were transformed with KpnI/SacI-digested plasmid and donor DNA, then selected on YPD medium containing 200 μg/ml nourseothricin. All strains were confirmed by PCR or sequencing. Primers used are listed in Table S1.

Fluorescence microscopy

To observe endocytosis, cells were stained with 8 μM of FM4-64 in YPD medium for 20 min and then chased in YPD medium for 20 min. After incubation, the cells were washed with PBS containing 20 mM NaN_3 and intracellular FM4-64 signal was examined using fluorescence microscopy (Nikon Eclipse E1 microscope with a Plan Fluor 100×/1.30 NA oil immersion objective, Nikon).

For fluorescence labeling of PPNPs, 5 mg of FITC and 50 mg of lyophilized PP were dissolved in 1 ml of DMF and stirred at 4℃ in the dark overnight. Subsequently, the reaction mixture was dropped into ethanol to remove the unreacted FITC and then centrifuged. The collected FITC-PPNP pellet was dried, dissolved in 1 ml of DMF, and dialyzed against distilled water at 4℃ for 48 h.

To observe the internalization of FITC-PPNPs, *C. albicans* cells in the logarithmic phase were harvested and resuspended in SC medium containing 1 mg/ml FITC-PPNPs, and incubated at 37℃ for 1 h. After incubation, the cells were washed with PBS and observed using fluorescence microscopy or analyzed by flow cytometry (BD FACS Canto II flow cytometer, Becton Dickinson).

Monitoring fluconazole susceptibility of *C. albicans*

SC medium supplemented with 10% FBS and the appropriate concentrations of fluconazole and PPNPs was prepared. A total of 100 µl of the medium was added to each well of a 96-well cell culture plate, and *C. albicans* cells were inoculated at 100 cells per well. After incubation at 37℃ for 24 h, the cells were diluted, plated on YPD plates, and CFUs were counted. Viability was calculated using the condition without fluconazole and PPNPs as the 100% reference. Assays were conducted three times in duplicate.

Table S1. Oligonucleotides used in this study.

For quantitative real-time reverse transcription-PCR		
**Gene**	**Forward primer**	**Reverse primer**
YWP1	AAATGTCAAGAAACCACCG	AGTTTCACCTTGAGTTGGG
HWP1	ATTCCAAATATTCCAACTG	TTCTGAAGTGGTAGCTAAA
ALS3	CAACTTGGGTTATTGAAACAAAAACA	AGAAACAGAAACCCAAGAACAACCT
HYR1	ATGAAGGCTCTAACCATGG	CAGAGCCATCGTTATGACC
IHD1	CACCAATACCGCATCCACT	GTAGAAGAGCCTGAACCCG
ECE1	CCAGAAATTGTTGCTCGTGTTG	CAGGACGCCATCAAAAACG
SAP5	CAGAATTTCCCGTCGATGAGA	CATTGTGCAAAGTAACTGCAACAG
SAP6	GGATTCCAGACTCAAAAGCCA	GTTCTGGTAGCTTCGTTGGC
HSP12	TGTTGGCTCAAATGTTCCAG	TTCAGCAGCCTTTCCAATTT
CTA1	ACTCCAGTGTTTTTCATTAGAG	AGAGTAACCATTCATTTCTCTG
SOD2	TTTCAACGGTGGTGGTTACC	ATAAAAGCCCATCCAGAACC
GPD1	AGTATGTGGAGCTTTACTGGGA	CAGAAACACCAGCAACATCTTC
**For gene disruption**		
END3-sg	ATTTGATTTCTGATTCTTCTAAACGG	AAAACCGTTTAGAAGAATCAGAAATC
END3-Donor	CTCACAGCCAACAAACCAAAAACTTTCATTATCGTTGAAACCGAAATAGCTCGAGCCACC	GTTTTACTGAAGATAAATCAGTAGAATTTTTGCTGGCGTTGGTGGCTCGAGCTATTTCGG
END3-CHK	ATAAAGGTCGTGTGCTTTGTGT	GTTCTGGATGTTACTGCTG
SNF7-sg	ATTTGAACTTGAATAGTAAACATGTG	AAAACACATGTTTACTATTCAAGTTC
SNF7-Donor	CCCGGAAATACATCCAAATCAAAACGACAAATCAACACCAGGTTCTAGCTCGAGAATGGG	CGTTATTCTCCGTATTCGGTATTTCAAACACATCATAATCCCATTCTCGAGCTAGAACC
SNF7-CHK	ACATGGATTGGTCGTGTAAC	TGTTATCAAAAAACAGGGGC

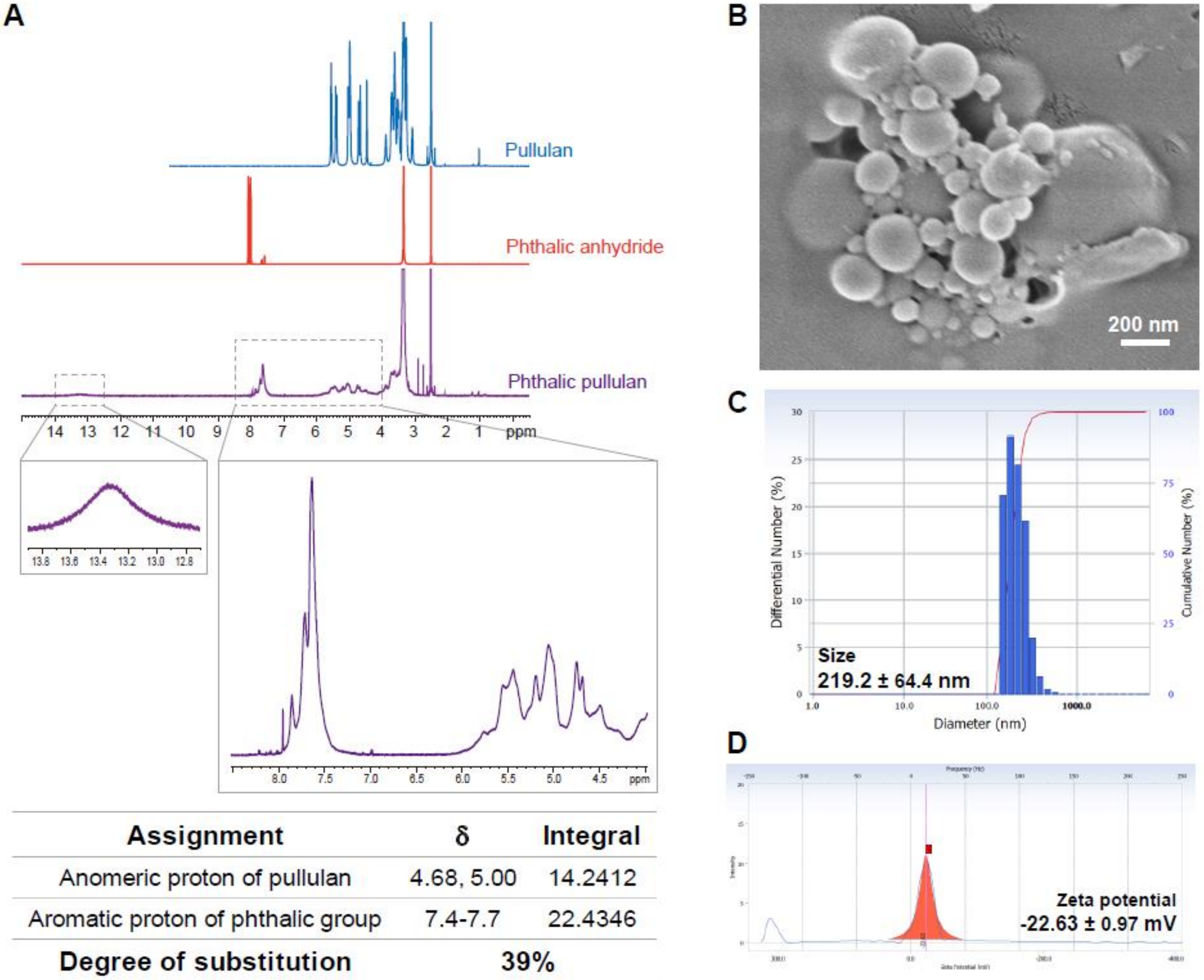

Assignment	δ	Integral
Anomeric proton of pullulan	4.68, 5.00	14.2412
Aromatic proton of phthalic group	7.4-7.7	22.4346
**Degree of substitution**	**39%**	

Figure S1. Characteristics of phthalic pullulan nanoparticles (PPNPs). (A) Calculation of the degree of substitution of phthalic moieties within phthalic pullulan by ^{1}H-NMR spectroscopy. (B) Morphology of PPNPs observed by FE-SEM. (C) Measurement of the size of PPNPs by DLS. (D) Measurement of the zeta potential of PPNPs by ELS. For (C) and (D), each value represents the mean ± standard deviation.

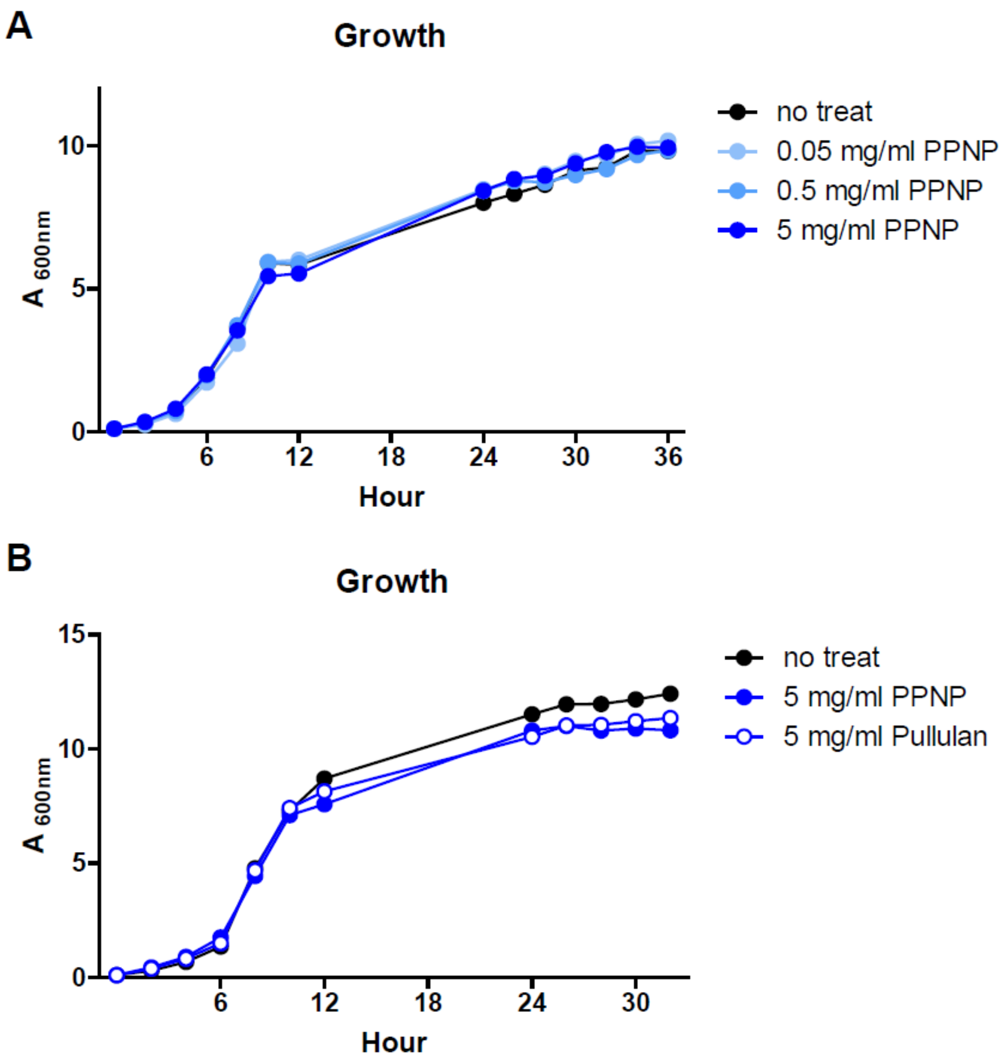

Figure S2. The effect of PPNPs on the growth of *C. albicans*. Growth curve of *C. albicans* cells under PPNP treatment conditions. *C. albicans* ATCC10231 cells were incubated at 37℃ in SC medium with the indicated concentrations of PPNPs (A), or with 5 mg/ml of PPNPs or pullulan (B).

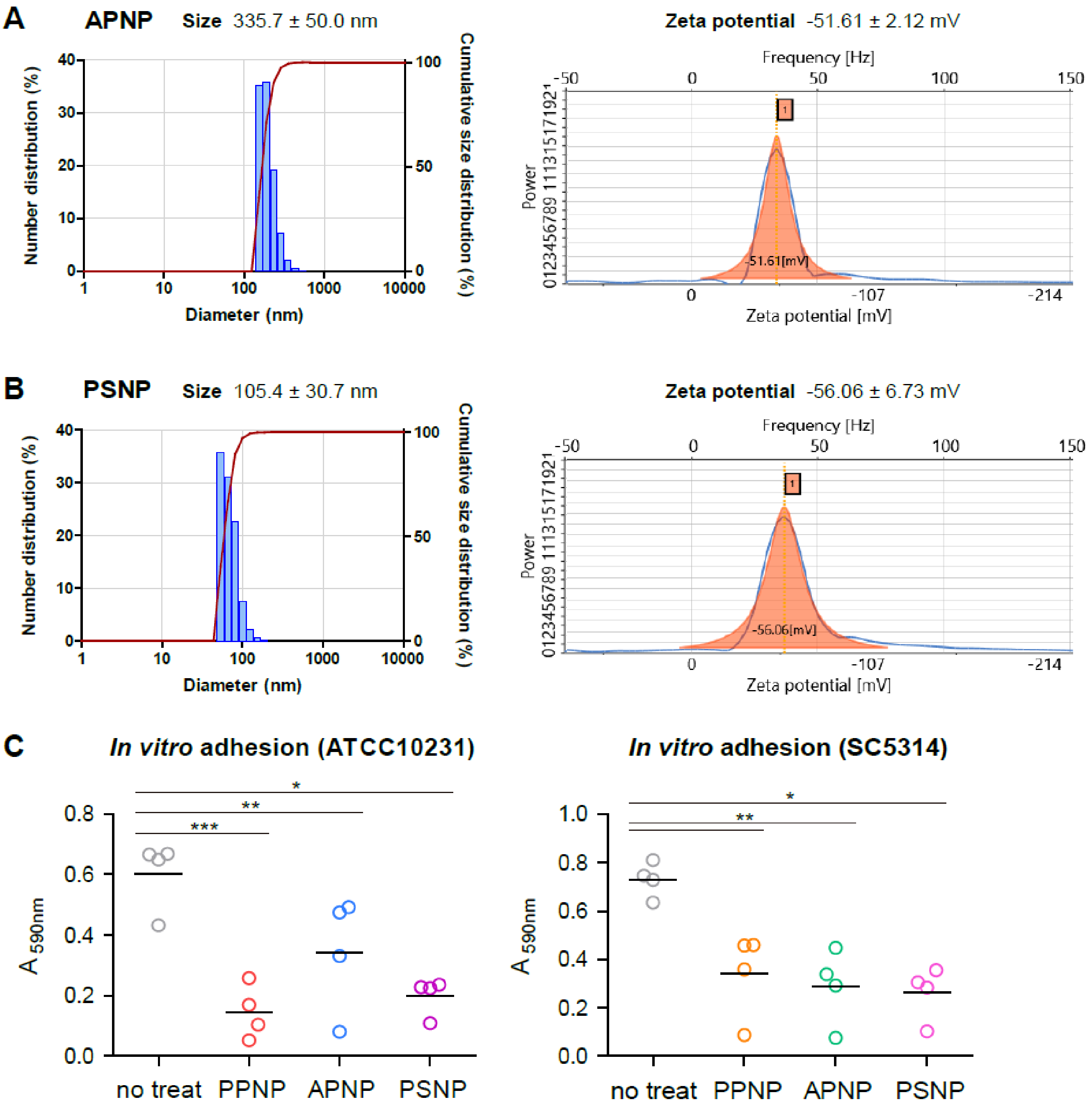

Figure S3. Inhibitory effect of various NPs on *C. albicans* adhesion to abiotic surfaces. (A-B) Measurement of the size (left panel) and the zeta potential (right panel) of APNPs (A) and PSNPs (B) by DLS and ELS. Each value represents the mean ± standard deviation. (C) *C. albicans* ATCC10231 cells or SC5314 cells were incubated at 37℃ for 1 h in SC medium containing 10% FBS with 5 mg/ml of indicated NPs. Cell adhesion was quantified by crystal violet staining. Asterisks indicate significant differences (paired two-tailed Student's *t*-test): *$P < 0.05$; **$P < 0.01$; ***$P < 0.001$.

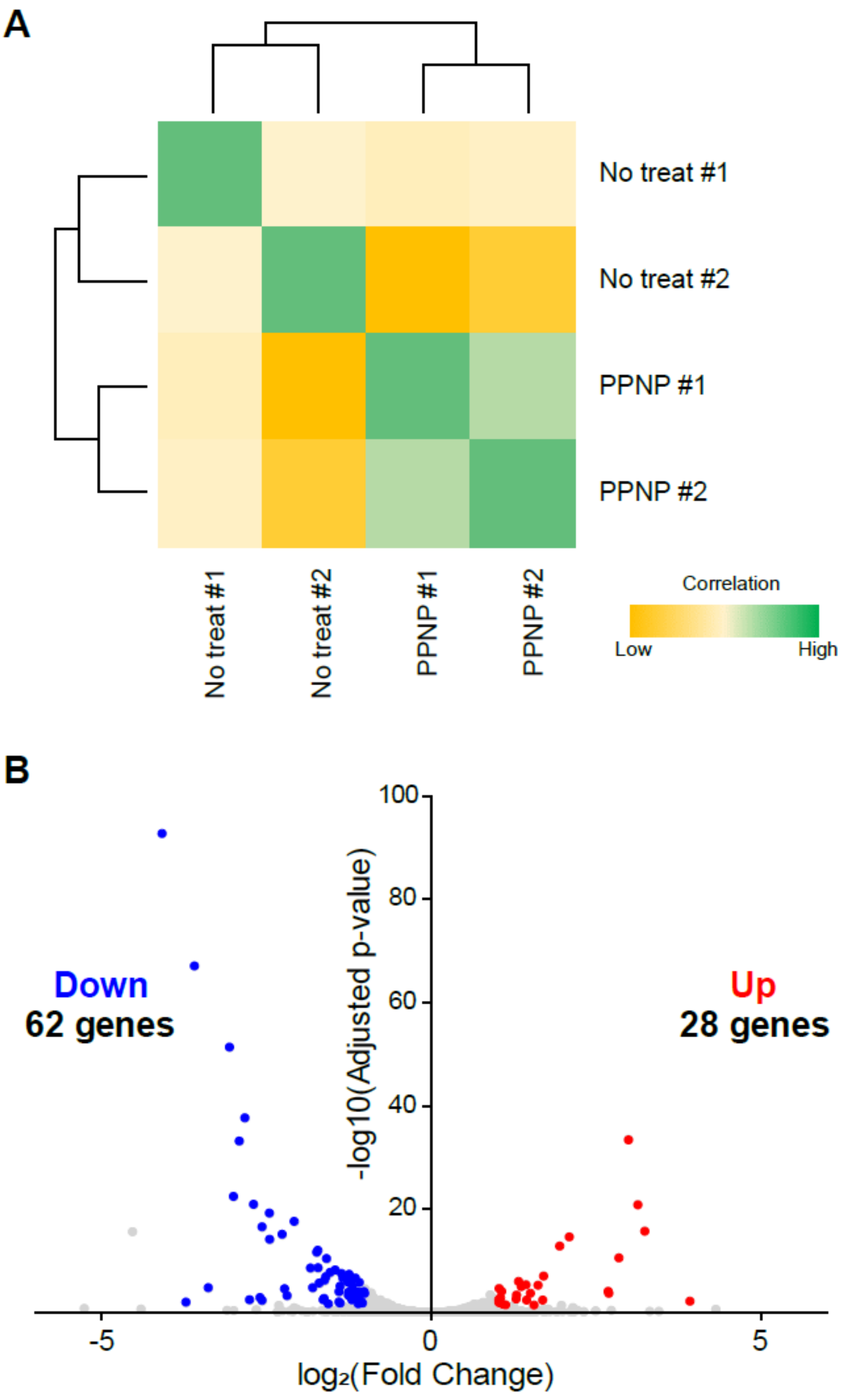

Figure S4. Global transcriptomic change of the *C. albicans* SC5314 response to treatment with 5 mg/ml PPNPs. *C. albicans* SC5314 cells cultured with or without 5 mg/ml PPNPs in SC medium supplemented with 10% FBS at 37℃ for 2 h. (A) Hierarchical clustering heat map of gene expression data. (B) Volcano plot representing upregulated (red) and downregulated (blue) genes in samples treated with 5 mg/ml PPNPs. The cutoff values are a fold change > 2 and a Benjamini-Hochberg adjusted *P*-value < 0.05.

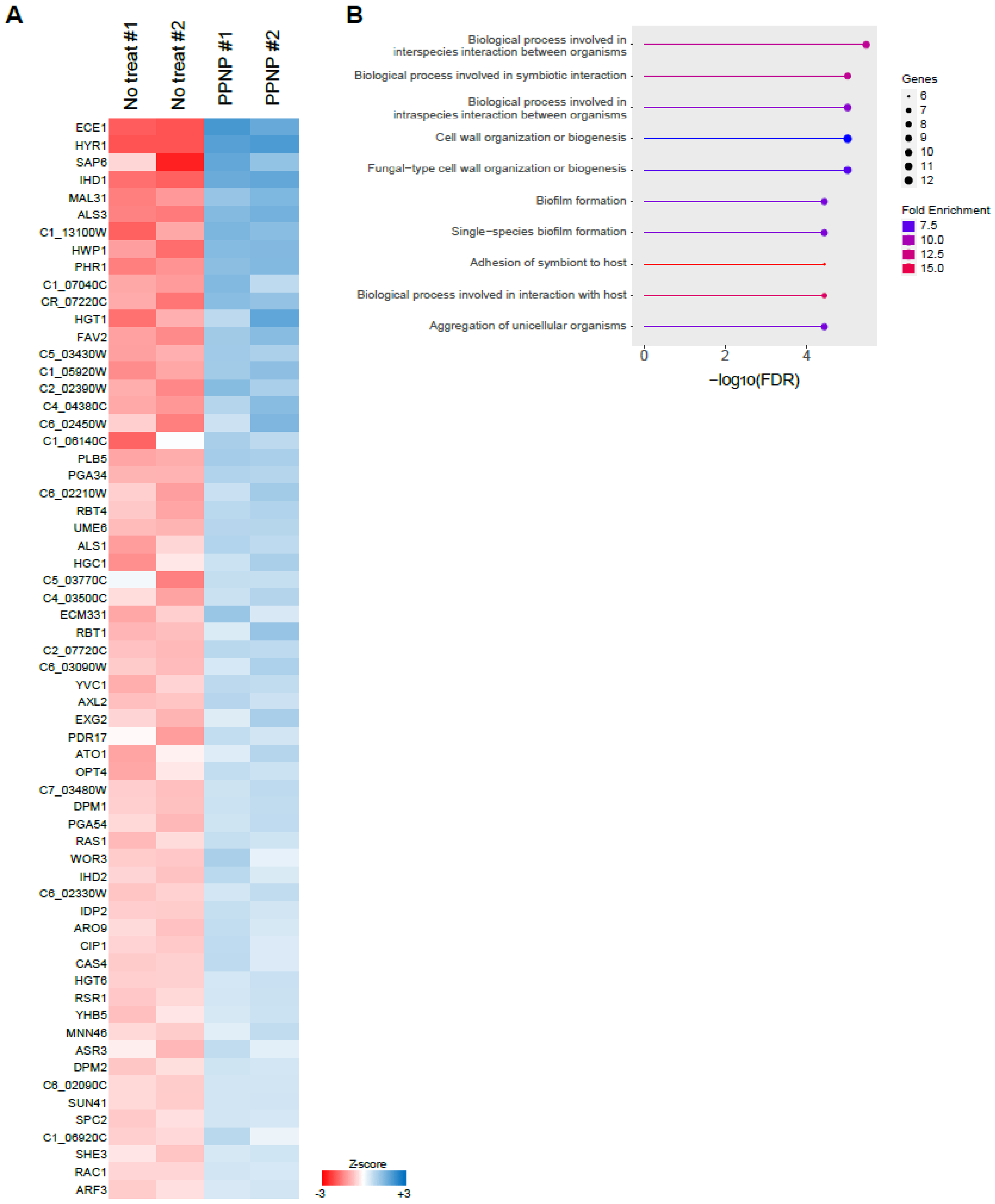

Figure S5. Downregulated genes and overrepresented GO terms for biological processes downregulated in response to treatment with 5 mg/ml PPNPs. (A) Heat map representing 62 downregulated genes in samples treated with 5 mg/ml PPNPs. (B) Top 10 significantly enriched GO terms for biological processes associated with downregulated genes in these samples. The cutoff value for significance is a false discovery rate (FDR) < 0.05.

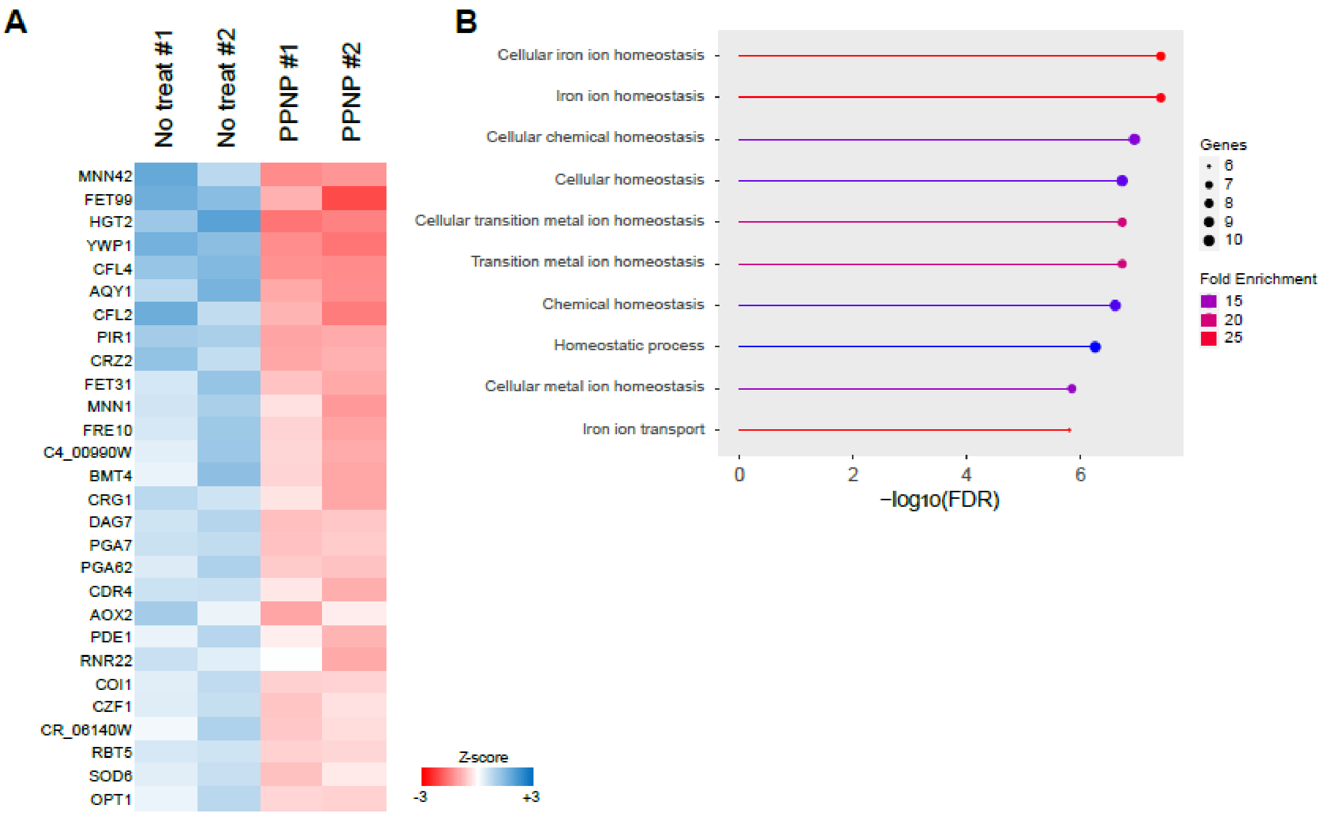

Figure S6. Upregulated genes and overrepresented GO terms for biological processes upregulated in response to treatment with 5 mg/ml PPNPs. (A) Heat map representing 28 upregulated genes in samples treated with 5 mg/ml PPNPs. (B) Top 10 significantly enriched GO terms for biological processes associated with upregulated genes in these samples. The cutoff value for significance is a false discovery rate (FDR) < 0.05.

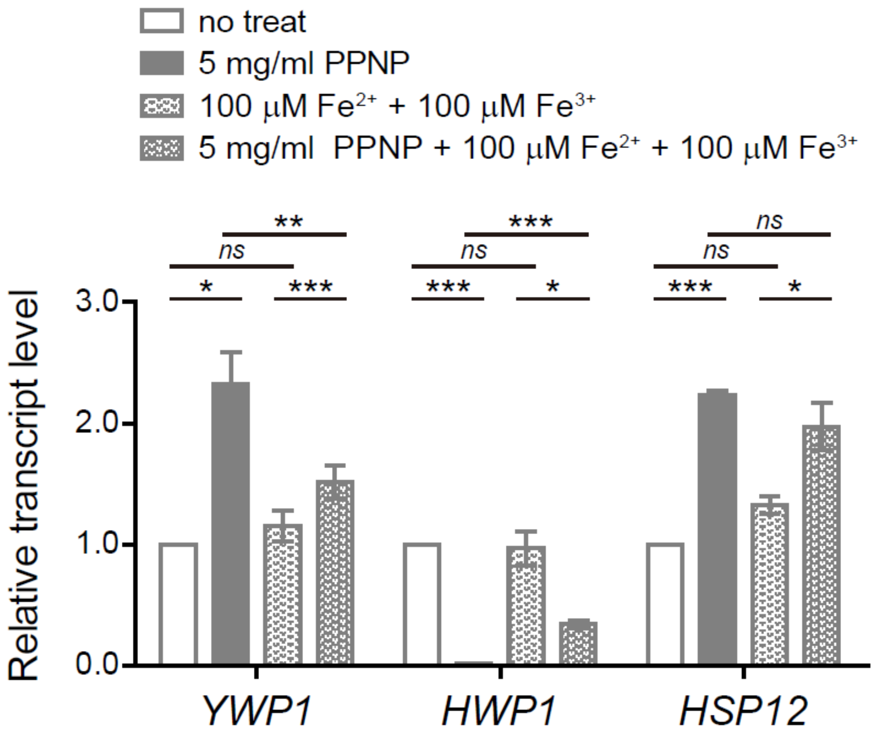

Figure S7. Supplementation with iron ion reduces the inhibitory effect of PPNPs on hypha-related gene expression of *C. albicans* cells. *C. albicans* SC5314 cells were incubated in SC medium containing 10% FBS with 5 mg/ml of PPNPs, 100 μM $FeSO_4$, and 100 μM $FeCl_3$ at 37℃ for 1 h. Total RNA was extracted, and the transcript levels were measured by qRT-PCR. Amplification efficiencies were validated and normalized against *GPD1*. Asterisks indicate significant differences (paired two-tailed Student's *t*-test): *$P < 0.05$; **$P < 0.01$; ***$P < 0.001$; ns, not significant.

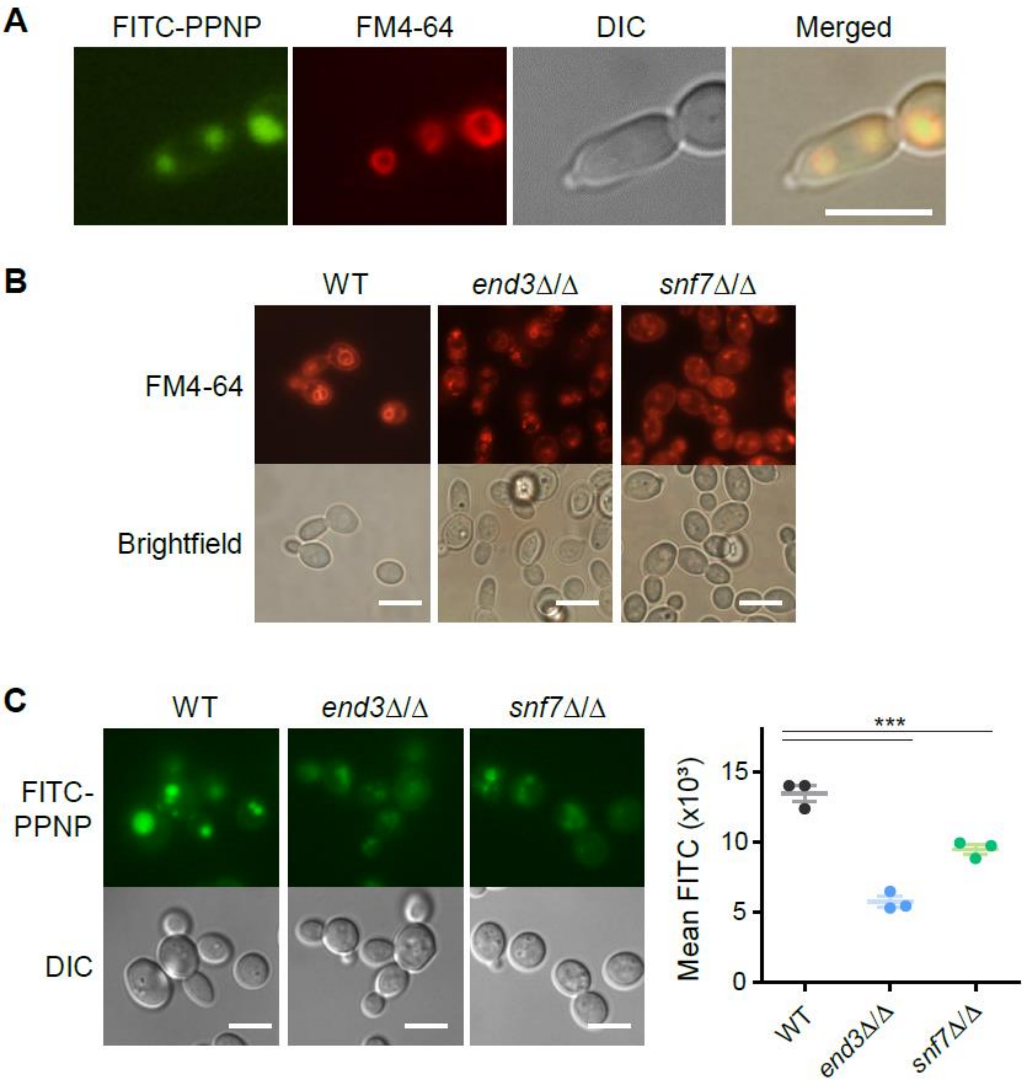

Figure S8. PPNPs are internalized into *C. albicans* cells through endocytosis. (A) Internalization of FITC-PPNPs into *C. albicans* cells. Cells were treated with 1 mg/ml FITC-PPNPs at 37℃ for 1 h. Vacuolar membranes were stained with 8 μM FM4-64 for 20 min. (B) Validation of the endocytosis-defective *end3*Δ/Δ and *snf7*Δ/Δ mutants by FM4-64 staining using fluorescence microscopy. Cells were stained with 8 μM FM4-64 in YPD medium for 20 min and then chased in YPD medium for an additional 20 min. (C) Examination of FITC-PPNP endocytosis using fluorescence microscopy and flow cytometry. Cells were treated with 1 mg/ml of FITC-PPNPs in SC medium for 1h. Asterisks indicate significant differences (paired two-tailed Student's *t*-test): ***$P < 0.001$. Scale bars: 5 μm.

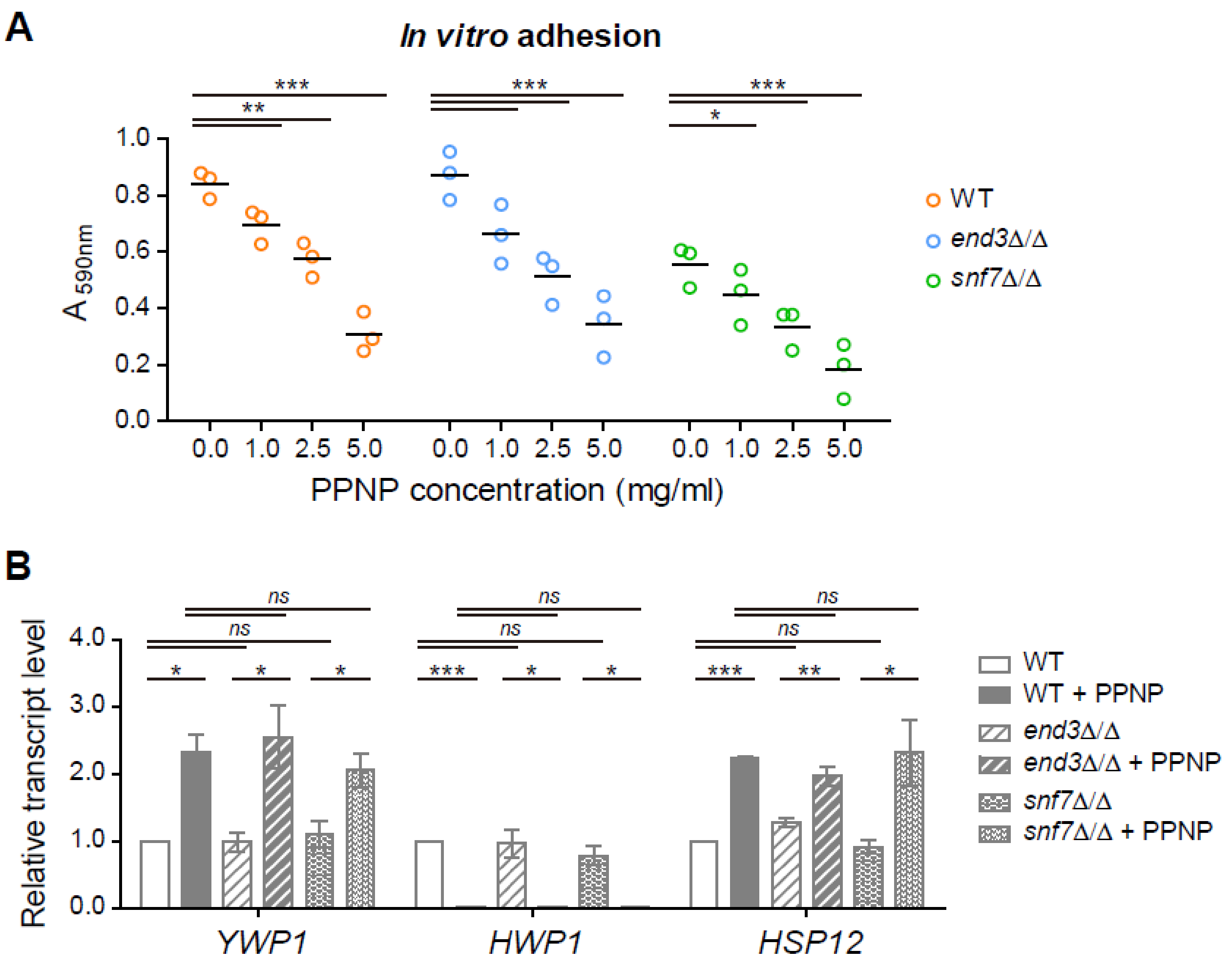

Figure S9. The inhibitory effect of PPNPs on *C. albicans* cells is independent of endocytosis. (A) *In vitro* adhesion of endocytosis-defective mutants. Cells were incubated in SC medium containing 10% FBS with the indicated concentrations of PPNPs at 37℃ for 1 h. Adherent cells were quantified by crystal violet staining. (B) PPNP-induced transcriptional regulation in endocytosis-defective mutants. Cells were incubated in SC medium containing 10% FBS with 5 mg/ml of PPNPs for 1 h. Total RNA was extracted, and the transcript levels were measured by qRT-PCR. Amplification efficiencies were validated and normalized against *GPD1*. Asterisks indicate significant differences (paired two-tailed Student's *t*-test): *$P < 0.05$; **$P < 0.01$; ***$P < 0.001$; ns, not significant.

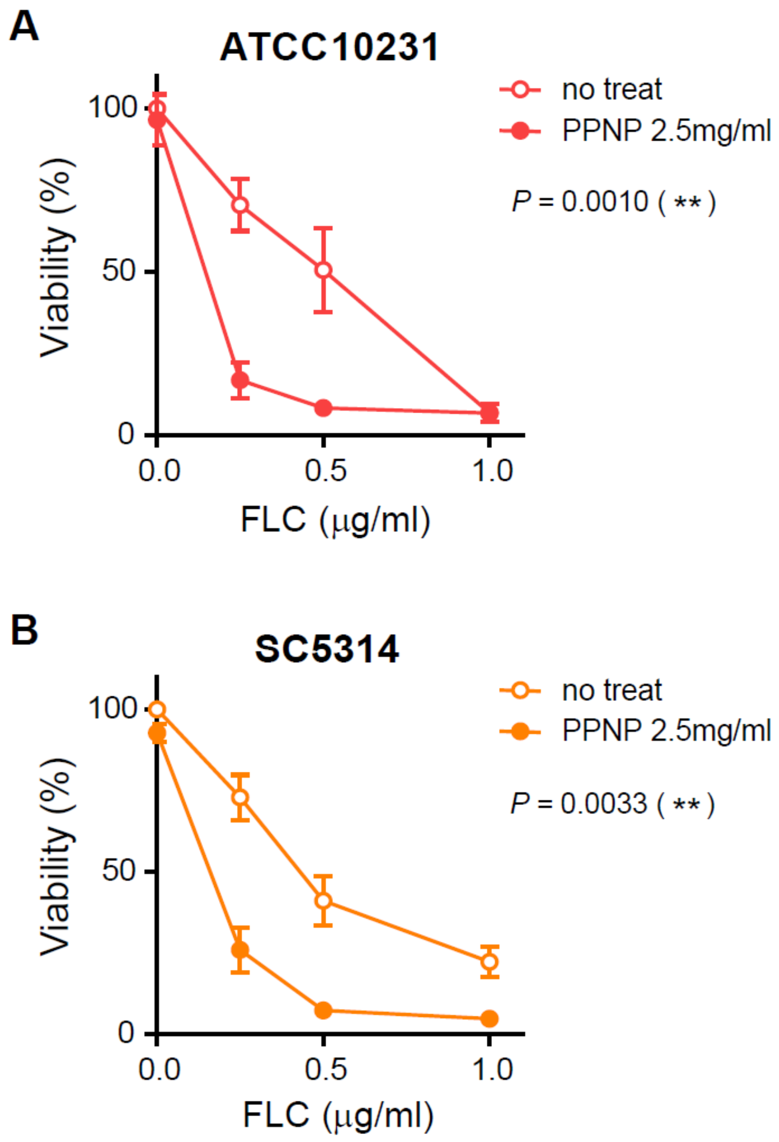

Figure S10. PPNPs increase the susceptibility of *C. albicans* to fluconazole. (A and B) *C. albicans* ATCC10231 cells (A) and SC5314 cells (B) were incubated in SC medium containing 10% FBS with the indicated concentrations of fluconazole (FLC) and PPNPs at 37℃ for 24 h. Viability was calculated by counting colony-forming units, with the condition without FLC and PPNPs used as the baseline (100%). Asterisks indicate significant differences (Two-way ANOVA): $^{**}P < 0.01$.

References

1. Ares M. 2012. Isolation of total RNA from yeast cell cultures. Cold Spring Harbor Protocols 2012:pdb. prot071456.
2. Dobin A, Davis CA, Schlesinger F, Drenkow J, Zaleski C, Jha S, Batut P, Chaisson M, Gingeras TR. 2013. STAR: ultrafast universal RNA-seq aligner. Bioinformatics 29:15-21.
3. Ge SX, Son EW, Yao R. 2018. iDEP: an integrated web application for differential expression and pathway analysis of RNA-Seq data. BMC Bioinformatics 19:534.
4. Ashburner M, Ball CA, Blake JA, Botstein D, Butler H, Cherry JM, Davis AP, Dolinski K, Dwight SS, Eppig JT, Harris MA, Hill DP, Issel-Tarver L, Kasarskis A, Lewis S, Matese JC, Richardson JE, Ringwald M, Rubin GM, Sherlock G. 2000. Gene ontology: tool for the unification of biology. The Gene Ontology Consortium. Nat Genet 25:25-9.
5. Gene Ontology C, Aleksander SA, Balhoff J, Carbon S, Cherry JM, Drabkin HJ, Ebert D, Feuermann M, Gaudet P, Harris NL, Hill DP, Lee R, Mi H, Moxon S, Mungall CJ, Muruganugan A, Mushayahama T, Sternberg PW, Thomas PD, Van Auken K, Ramsey J, Siegele DA, Chisholm RL, Fey P, Aspromonte MC, Nugnes MV, Quaglia F, Tosatto S, Giglio M, Nadendla S, Antonazzo G, Attrill H, Dos Santos G, Marygold S, Strelets V, Tabone CJ, Thurmond J, Zhou P, Ahmed SH, Asanitthong P, Luna Buitrago D, Erdol MN, Gage MC, Ali Kadhum M, Li KYC, Long M, Michalak A, Pesala A, Pritazahra A, Saverimuttu SCC, et al. 2023. The Gene Ontology knowledgebase in 2023. Genetics 224.
6. Thomas PD, Ebert D, Muruganujan A, Mushayahama T, Albou LP, Mi H. 2022. PANTHER: Making genome-scale phylogenetics accessible to all. Protein Science 31:8-22.
7. Evans BA, Pickerill ES, Vyas VK, Bernstein DA. 2018. CRISPR-mediated Genome Editing of the Human Fungal Pathogen Candida albicans. J Vis Exp doi:10.3791/58764.
8. Vyas VK, Bushkin GG, Bernstein DA, Getz MA, Sewastianik M, Barrasa MI, Bartel DP, Fink GR. 2018. New CRISPR mutagenesis strategies reveal variation in repair mechanisms among fungi. MSphere 3:10.1128/msphere. 00154-18.
9. Labun K, Montague TG, Krause M, Torres Cleuren YN, Tjeldnes H, Valen E. 2019. CHOPCHOP v3: expanding the CRISPR web toolbox beyond genome editing. Nucleic Acids Res 47:W171-W174.
